# A Hybrid Approach for CpG Island Detection in the Human Genome

**DOI:** 10.1371/journal.pone.0144748

**Published:** 2016-01-04

**Authors:** Cheng-Hong Yang, Yu-Da Lin, Yi-Cheng Chiang, Li-Yeh Chuang

**Affiliations:** 1 Department of Electronic Engineering, National Kaohsiung University of Applied Sciences, Kaohsiung, Taiwan; 2 Department of Chemical Engineering & Institute of Biotechnology and Chemical Engineering, I-Shou University, Kaohsiung, Taiwan; The George Washington University, UNITED STATES

## Abstract

**Background:**

CpG islands have been demonstrated to influence local chromatin structures and simplify the regulation of gene activity. However, the accurate and rapid determination of CpG islands for whole DNA sequences remains experimentally and computationally challenging.

**Methodology/Principal Findings:**

A novel procedure is proposed to detect CpG islands by combining clustering technology with the sliding-window method (PSO-based). Clustering technology is used to detect the locations of all possible CpG islands and process the data, thus effectively obviating the need for the extensive and unnecessary processing of DNA fragments, and thus improving the efficiency of sliding-window based particle swarm optimization (PSO) search. This proposed approach, named ClusterPSO, provides versatile and highly-sensitive detection of CpG islands in the human genome. In addition, the detection efficiency of ClusterPSO is compared with eight CpG island detection methods in the human genome. Comparison of the detection efficiency for the CpG islands in human genome, including sensitivity, specificity, accuracy, performance coefficient (*PC*), and correlation coefficient (*CC*), ClusterPSO revealed superior detection ability among all of the test methods. Moreover, the combination of clustering technology and PSO method can successfully overcome their respective drawbacks while maintaining their advantages. Thus, clustering technology could be hybridized with the optimization algorithm method to optimize CpG island detection.

**Conclusion/Significance:**

The prediction accuracy of ClusterPSO was quite high, indicating the combination of CpGcluster and PSO has several advantages over CpGcluster and PSO alone. In addition, ClusterPSO significantly reduced implementation time.

## Introduction

CpG islands are short sequences in a genome, with high concentrations of Cytosine (C) and Guanine (G) nucleotides where CpG islands include CpG dinucleotides (CpGs). Gardiner-Garden and Frommer [1] defined CpG islands as having the following properties: (i) the length of the island region exceeds 200 bps, (ii) GC content is higher than 50%, and (iii) CpG frequency (observed/expected, O/E) surpasses 0.6. In 2002, Takai and Jones proposed a rigorous CpG island definition [2], including a minimum length of 500 bps, GC content of 55% and an O/E ratio of 0.65. The 500 bp length is proposed to avoid *Alu* sequences in CpG islands. An *Alu* sequence indicates a highly repetitive short interspersed element with an approximate consensus sequence of about 280 bps, and the sequence exhibits high GC content levels and O/E ratio.

DNA methylation is a chemical modification of DNA which has been reported to potentially affect gene transcription and may regulate long-term memory storage in humans [3]. About 80% of all CpGs have been found to be methylated in human and mouse genomes, and some methylated CpGs are located in CpG islands [4]. Approximately 60% of all genes have a CpG island in their promoter region, and these promoter-associated CpG islands may undergo methylation to change their gene expression (the promoter regions contain about 70% of CpG islands in human genes). For example, abnormal cancer cells can cause hypermethylation in promoter CpG islands and thereby contribute to the development of cell lesions and drug resistance [5–7]. Thus, development of an accurate method for CpG island detection could be useful in research for drug, cancer, and genomic markers.

Current CpG island detection methods are mainly based on the Gardiner-Garden and Frommer (GGF) definition [1], e.g., CpGProD [8], CpGIS [2], CpGplot [9], and particle swarm optimization (PSO)-based methods [10]. These methods use a broad range of CpG island properties, including GC content, O/E ratio and length thresholds, and employ the sliding window approach to scan DNA sequences for CpG island detection. The CpGProD and CpGplot methods are similar to CpGIS in which the extraction process is divided into four steps. Firstly, a 200 bp window is set in the beginning of the sequence, and then checked to determine whether the window meets the CpG island criteria. If the window does not meet the criteria, the window is moved 1 bp toward the 3' until the window meets the CpG island criteria. If the window does meet the criteria, the window is shifted 200 bps and evaluated again. This step is repeated until the window does not meet the criteria. Thirdly, the last window is moved 1 bp toward the 5' until it meets the criteria. Finally, the total GC content and O/E ratio is calculated to determine whether the large CpG island meets the criteria. If the CpG island does not meet the criteria, then 1 bp is trimmed from the beginning and ending of the sequence until it meets the criteria. Moreover, if two individual CpG islands are separated by a distance of less than 100 bps, then these two islands are combined and the GC content and O/E ratio are recalculated such that they adhere to the criteria. In addition, Chuang *et al*. proposed a new PSO-based method, in which an input sequence is divided into multiple 2,500 bp windows due to the length of the chromosome sequences. Each window is subjected to PSO for CpG island prediction. This method provides simple and accurate CpG island detection with fast convergence and fewer parameters. However, PSO using the sliding window approach to scan DNA sequences is time-consuming. The CpGcluster method is a CpG island detection technique [11] that directly uses statistical properties to detect CpG dinucleotide clusters without considering CpG island definitions. The use of integer arithmetic allows for the quick detection of statistically-significant CpG dinucleotide clusters in which CpG islands have a higher degree of overlap with promoter regions and highly-conserved elements [12]. However, the sensitivity of the CpGcluster is low because of its short length, high O/E ratio and high GC content [13].

This study combines clustering technology (CpGcluster) and PSO to develop a simple and accurate method (ClusterPSO) for detecting CpG islands. CpGcluster is first introduced in the pre-treatment strategies for detecting all CpG island candidates; these CpG islands are evaluated according to the distances between CpGs and their *p*-values (*P* ≤ 0.01). PSO is then used to predict CpG islands from all CpG island candidates obtained from the CpGcluster results. The GGF criteria are used as a basis to compare results with other methods based on five evaluation criteria including sensitivity, specificity, accuracy, performance coefficient (*PC*), and correlation coefficient (*CC*). Results showed that ClusterPSO provides high efficiency for CpG island detection with improved sensitivity, accuracy, and correlation coefficient, while reducing search time in the human genome.

## Methods

### CpGcluster

CpGcluster was first proposed by Hackenberg *et al*. [11]. The basic theory of CpGcluster is based on the physical distance between neighboring CpGs to directly detect CpG clusters without considering subjective CpG criteria. The CpGcluster procedure has two parts: (i) a distance-based algorithm searches for CpG clusters in the genome, and (ii) the *p*-value criteria selects the statistically significant CpG clusters. The detailed procedure is explained in the ClusterPSO description below.

### Particle swarm optimization

In PSO, each particle represents a practicable solution *x*_*i*_ = {*x*_*i1*_, *x*_*i2*_, …, *x*_*id*_} in the *d*-dimensional problem space, and it has a memory function to accumulate its own experience defined as the *pbest*_*i*_ = {*pbest*_*i1*_, *pbest*_*i2*_, …, *pbest*_*id*_} [14]. All particles share a common memory used to identify the current best experience *gbest* = {*gbest*_1_, *gbest*_2_, …, *gbest*_*d*_}. Based on these two experiences, a particle moves to find a better solution. Since each particle adopts a directional trajectory in the search process, it can effectively find a better solution by continually updating its location. The PSO procedure includes five steps: (i) initialization, (ii) fitness evaluation, (iii) updating the *pbest*_*i*_ and *gbest*, (iv) updating the positions and velocities of particles, and (v) confirmation that the stop criterion is met. All steps are explained in detail in the ClusterPSO section.

### ClusterPSO

The proposed CpGcluster is used to detect all possible CpG island locations (CpG island candidates) and optimize data processing, thus increasing PSO effectiveness by removing huge amounts of unnecessary DNA fragments from the calculations. Since the known CpG island minimum length detected by CpGcluster is 8 bps [11], all CpG island candidates are extended both forward and backward by 200 bps to conform with the GGF criteria, where 200 bps is the known minimum CpG island length in the GGF criteria. This region of CpG island candidates allows the PSO to predict optimal CpG islands using the GGF criteria.

**Fig 1:**
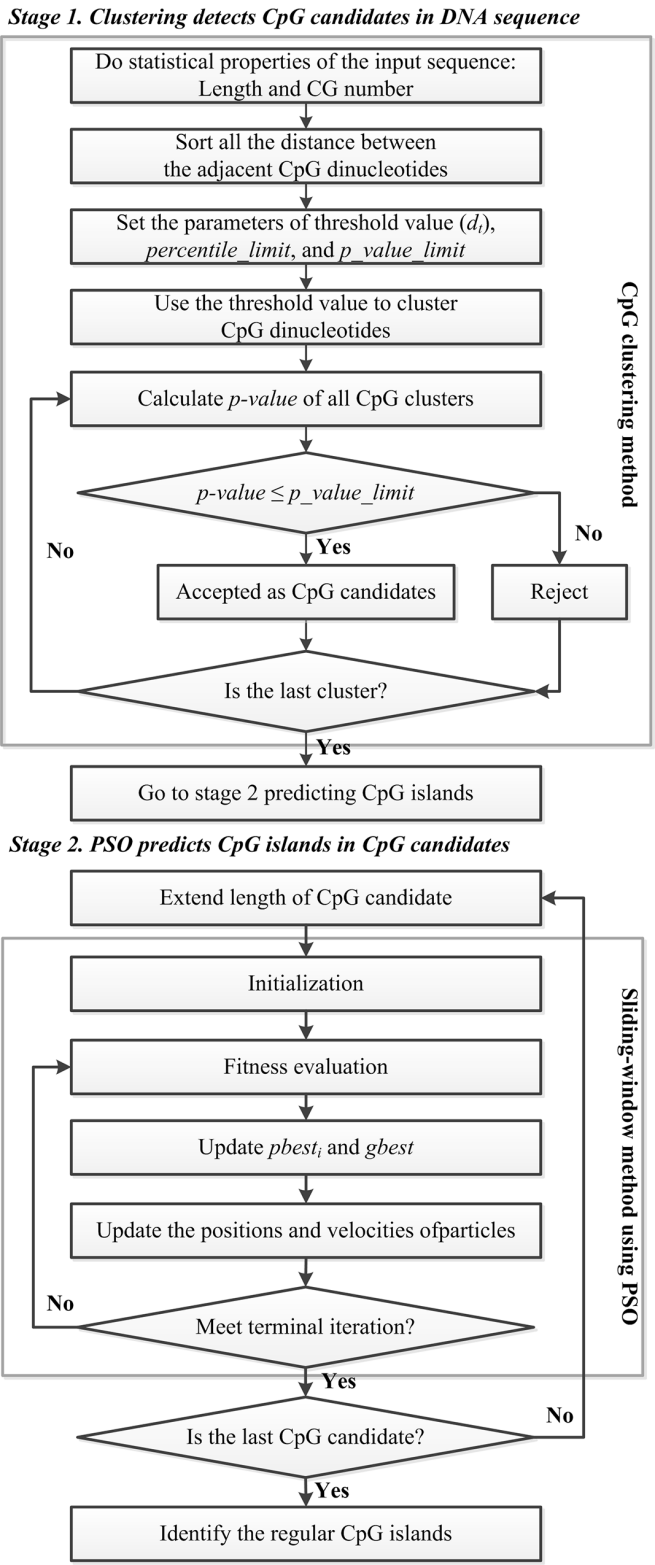
ClusterPSO Flowchart.

Fig 1 shows the ClusterPSO flowchart divided into two stages: 1. CpGcluster detects CpG island candidates in the DNA sequence. This stage has two sub-steps: (i) Clustering technology and statistical search methods are used to identify CpG island candidates in the chromosome sequence. The distance threshold is used to combine adjacent and sufficiently close CpG dinucleotides as clusters. (ii) The *p*-value is used to verify CpG island candidates. 2. PSO predicts CpG islands among the CpG island candidates based on the GGF relaxed CpG island criteria. Here we provide an example to illustrate how the ClusterPSO works, with details provided in the Additional file.

#### Stage 1. Clustering detects CpG candidates in DNA sequence

CpG clustering within the CpG island region is determined by a distance threshold, with closely adjacent CpGs belonging to the same cluster, and longer distances differentiating different clusters. Thus, the intensive CpGs are set into a cluster to select CpG island candidates. All steps are described in detail as follows:

Step 1. All CpG positions are scanned from 5' to 3' in a DNA sequence, and the CpG positions are collected into a set *C* = {*c*_1_, *c*_2_, …, *c*_*n*_}, where *n* is the total number of CpGs.
Step 2. The distances of all adjacent CpGs are calculated, in which a physical distance between two adjacent CpGs is computed by *d*_*i*_ = *X*_*i +1*_ –*X*_*i*_*−* 1. The shortest distance of adjacent CpGs is 1, i.e., CGCG.
Step 3. A threshold value (*d*_*t*_) is defined according to the distribution of distances of all adjacent CpGs in a DNA sequence, and used to determine whether the adjacent CpGs belong to a same cluster or not. The set *C* is sorted according to the distance between adjacent CpGs, and the threshold *d*_*t*_ is defined at the 65 percentile in the set *C*.
Step 4. CpGcluster uses a threshold value to start extending downstream (→ 3') from the first CpG of set *C*. When the adjacent distance between CpGs is smaller than the threshold value, the two adjacent CpGs are classified into a single cluster; otherwise, the position of the upstream G nucleotide of the adjacent CpGs is defined as the closing cluster position. Thus, step 4 continues to search for new clusters until it meets the last CpG.

#### p-value criteria selects the statistically significant CpG clusters

Since all CpG island clusters are determined by distance, the *p*-value of a cluster is then computed to calculate the probability of a CpG cluster appearing in a random sequence. The negative binomial distribution estimates the probability to reduce the requirements of the CpG clusters. The distribution fails if the number of successes is fixed in advance. Thus, the successes represent the CpGs and the failures represent non-CpGs. The above-mentioned probability is calculated by the cumulative density function at point *n*_*f*_ of the CpG cluster, and is taken as the *p*-value:

$$P_{N,p}^{cum}(x<=n_{f})=\sum_{x=0}^{n_{f}}\left(\begin{array}{c} x-(N+1)-1\\ (N-1)-1 \end{array}\right)\times p^{N-1}\times {\left(1-p\right)}^{x}$$ (1)

$$n_{f}=L-2\times N$$ (2)

$$p=\frac{N_{s}}{N_{is}}$$ (3)

where *N* is the number of CpGs in the cluster, *n*_*f*_ is the number of independent non-CpGs in the cluster, *L* is the number of nucleotides in the cluster, and *p* is the probability of success finding a CpG. *N*_*s*_ and *N*_*is*_ are the number of CpGs and the number of independent dinucleotides in the DNA sequence, respectively. This step searches statistically significant CpG clusters and assumes that all CpG islands are included in these CpG clusters. When the *p*-value of a cluster is smaller than the threshold value (set as 10^−2^ in this study), the clusters are discarded; otherwise, the cluster is retained.

#### Stage 2. PSO predicts CpG islands from CpG candidates

Since the CpGcluster step collects CpG island candidates without the CpG island criteria, the CpG island candidates may be shorter than 200 bp. The lengths of these CpG island candidates must be extended for the application of PSO prediction. All CpG island candidates are extended forward and backward by 200 bps, and a separate PSO procedure is used for each specific CpG island candidate, which can be divided into the following five steps:

Step 1. Initialization defines the particle as (population size = 300) a possible CpG island region, and the particle coordinates constitute a two-dimensional problem space, i.e., *d* = 2. Eq 4 is the particle encoding formula.
  $$P_{i}=\left(F_{si}+F_{li}\right)$$ (4)
  where *P*_*i*_ is the *i*^th^ particle. *Fs*_*i*_ and *Fl*_*i*_ are respectively the start position and length of a predicted CpG island in a CpG island candidate. At initialization, each particle *P*_*i*_ is randomly generated the parameters *Fs*_*i*_ and *Fl*_*i*_.
Step 2. Define the particle fitness by GGF criteria. The fitness function for evaluating the particles is based on the length, GC content, and O/E ratio of CpG island properties. A higher fitness value represents a better CpG island prediction result. In addition, the recorded particle positions must conform to the GGF definition for CpG island properties, such as the range of particle length ≥ 200 bps, GC content ≥ 50%, and O/E ratio ≥ 0.6. Then follow an overlap process to predict all CpG island locations. Eq 5 is the normalization function to calculate the CpG length. Eqs 6 and 7 respectively calculate the GC content and O/E ratio. Eq 8 is the fitness function.
  $$CpG_{len}(P_{i})=\frac{\#A+\#T+\#C+\#G}{p_{\max}-p_{\min}}$$ (5)
  $$GC(P_{i})=\frac{\#C+\#G}{\#A+\#T+\#C+\#G}$$ (6)
  $${\text{Obs}}_{\text{CpG}}{/\text{Exp}}_{\text{CpG}}\;(P_{i})=\frac{\frac{\#CpG}{CpG_{length}}}{\frac{\#C}{CpG_{length}}\times \frac{\#G}{CpG_{length}}}$$ (7)
  $$Fitness(P_{i})=GC(P_{i})+Obs_{CpG}/Exp_{CpG}(P_{i})+CpG_{len}(P_{i})$$ (8)
  where *#A*, *#T*, *#C*, and *#G* are respectively the numbers of nucleotide adenine (A), thymine (T), cytosine (C), and guanine (G) in the predicted CpG island region at *P*_*i*_. *p*_*min*_ is the starting position of the cluster minus 200, and *p*_*max*_ is the starting position of the cluster plus 200. *#CpG* represents the number of CpG in the predicted CpG island region at *P*_*i*_.
Step 3. Update the *pbest*_*i*_ and *gbest* for all particles. Each particle finds its personal best position (*pbest*) and the global best position (*gbest*) when moving. If the fitness value of a particle *P*_*i*_ in the current generation is higher than the fitness value of *pbest*, the fitness value and position of *pbest* is replaced by *P*_*i*_. If the fitness value of particle *P*_*i*_ in the current generation is higher than *gbest*, the fitness value and position of *gbest* is replaced by *P*_*i*_.
Step 4. Update the positions and velocities of particles in each generation. The position and velocity of the *P*_*i*_ is updated by the *pbest*_*i*_ and *gbest*. Eqs 9 and 10 respectively calculate the velocity and position of the updating formulas at *particle*_*i*_.
  $$v_{\;i,j}^{new}=w\times v_{i,j}^{old}+c_{1}\times r_{1}\times \left(pbest_{i,j}-x_{i,j}^{old}\right)+c_{2}\times r_{2}\times \left(gbest_{j}-x_{i,j}^{old}\right)$$ (9)
  $$x_{i,j}^{new}=x_{i,j}^{oid}+v_{i,j}^{new}$$ (10)
  where *r*_1_ and *r*_2_ are random variables between zero and one, and both *c*_1_ and *c*_2_ are constants. *c*_1_ and *r*_1_ account for the subjective experience of *particle*_*i*_ in the search process, while *c*_2_ and *r*_2_ account for the population experience. The *particle*_*i*_ velocities, $v_{id}^{new}$ and $v_{id}^{old}$ are respectively the new and old velocities. Positions $x_{id}^{new}$ and $x_{id}^{old}$ are respectively the updated and current positions.
  The inertia weight w is used to improve the particle search path. Using Eq 11, the inertia weight can be linearly decreased from 0.9 to 0.4 according to the generation number. Poli *et al*. [15] demonstrated how the inertia weight improved the balance between local search and global search to facilitate particle search.
  $$w=(w_{\max}-w_{\min})\times \frac{move_{\max}-move_{i}}{move_{\max}}+w_{\min}$$ (11)
  where *w*_max_ and *w*_min_ are respectively set at 0.9 and 0.4, and *move*_max_, *move*_*i*_ are respectively the maximum and current generations. Thus, each particle can adjust its direction based on a better *pbest* and *gbest* in the next generation.
Step 5. Confirm whether the stop criterion (maximum generation = 100) is met or not. Steps 2 to 5 are repeated until the maximum generation is reached. When all CpG island candidates are predicted by PSO, all the identified CpG islands represent the CpG island detection results.

#### Performance measurement

The measurement of association between binary variables, 2 × 2 contingency, is used to evaluate the accuracy of CpG island detection [16]. As shown in the contingency table, true positivity (*TP*) represents that the number of bases detected in the CpG island region are true CpG islands. True negative (*TN*) represents that the number of bases detected in the non-CpG island region is correct. False negative (*FN*) represents that the number of bases detected in the true CpG island region is incorrect, and false positive (*FP*) represents that some of the bases detected in the CpG island region are non-CpG islands. Five common criteria are used to evaluate the results, including accuracy, sensitivity, specificity, the performance coefficient (*PC*), and the correlation coefficient (*CC*). Eq 12 is used to evaluate prediction accuracy by comparing the detection results with the true CpG islands. Sensitivity is calculated by Eq 13 to compute the rate of correct CpG island detection. Eq 14 is used to evaluate the specificity for computing the proportion of correct non-CpG island detection. Eq 15 summarizes sensitivity and specificity as a measure of global accuracy. Eq 16 shows the correlation coefficient, which is a single scalar value for correct non-CpG island detection.

$$ACC=\frac{TP+TN}{TP+FP+TN+FN}$$ (12)

$$SN=\frac{TP}{TP+FN}$$ (13)

$$SP=\frac{TN}{TN+FP}$$ (14)

$$PC=\frac{TP}{TP+FP+FN}$$ (15)

$$CC=\frac{TP\times TN-FP\times FN}{\sqrt{\left(TP+FN)\times (TP+FP)\times (TN+FN)\times (TN+FP\right)}}$$ (16)

#### Parameter settings

In the CpGcluster step, the *p*-value and distance threshold (percentile) parameters are respectively set as 0.01 and 65^th^. In the PSO step, the parameters of population size, maximum generation, and both *c*_1_ and *c*_2_ are respectively set as 300 [17], 100, and 2 [15]. GGF [1] is used to define CpG islands to allow for comparisons with other GGF-based methods.

## Results

### Data set

In previous study [10], six regular contig sequences, including NT_113953.1, NT_113954.1, NT_113955.2, NT_113958.2 and NT_113952.1 in chromosome 21, and NT_028395.3 in chromosome 22, are selected to evaluate the performance of ClusterPSO and other methods. All contig sequences and CpG islands were verified based on sequence analysis and bisulphite sequencing (BS-seq) and were obtained from NCBI (http://www.ncbi.nlm.nih.gov), along with the entire human genome (NCBI.36). Eight CpG island detection methods, including CpGPlot, CpGcluster, CpGProD, CpGIS, and PSO-based methods, were selected for comparison with the proposed ClusterPSO method. All data sets and ClusterPSO source code were published on github (url: https://github.com/jackmel030/ClusterPSO).

### CpG island detection of contig sequences

Table 1 shows the results of six contig sequences detected by the nine test methods. Both ClusterPSO and CPSORL show a higher sensitivity, *PC*, and *CC* values in the six contig sequences. CpGPlot shows excellent SP results in all the contig sequences. CPSORL performs better than the methods of CpGPlot, CpGcluster, CpGProD, CpGIS, PSO, PSORL, and CPSO, but ClusterPSO outperforms CPSORL for sensitivity, accuracy, *PC*, and *CC* values in the six contig sequences. A non-parameter statistical hypothesis test, Wilcoxon Signed-Rank test, is used on pairs of result groups to gauge their validity. The *p*-value of the Wilcoxon Signed-Rank test can determine whether the difference between the two methods is significant or not. A *p*-value < 0.001 indicates the ClusterPSO is statistically significant and exhibits improved CpG island detection. All *p*-values for ClusterPSO *vs*. the other eight methods on the six contig sequence results result in *p* < 0.0001. Although some methods use different CpG island definitions, the 2 × 2 contingency seems to provide a higher accuracy for CpG island detection based on the known positions of CpG islands in the NCBI. The results of the nine methods are taken from the relevant literature.

**Table 1:** Comparison of different CpG island detection methods.

		Methods								
Contig		CpGPlot	CpGcluster	CpGProD	CpGIS	PSO	PSORL	CPSO	CPSORL	ClusterPSO
NT_113952.1	SN	56.43	50.46	58.07	83.98	69.22	75.58	77.43	84.88	**95.98**
	SP	**100.0**	99.95	99.50	99.05	99.61	99.02	99.58	99.05	99.47
	ACC	98.09	97.78	97.69	98.39	98.28	97.99	98.61	98.43	**99.32**
	PC	56.42	49.92	52.36	69.59	63.77	62.27	70.91	70.34	**86.16**
	CC	74.38	69.41	68.83	81.25	77.66	75.71	82.49	81.8	**92.28**
NT_113955.2	SN	47.19	67.15	68.51	85.12	54.47	59.63	77.8	87.38	**94.67**
	SP	**100.0**	99.72	99.63	99.30	99.96	99.88	99.5	99.61	99.51
	ACC	98.08	98.54	98.50	98.79	98.31	98.42	98.71	99.16	**99.33**
	PC	47.14	62.47	62.35	71.78	53.87	57.74	68.67	79.08	**83.81**
	CC	67.94	77.03	76.65	82.96	72.41	74.51	80.85	87.89	**90.92**
NT_113958.2	SN	51.29	27.16	46.41	82.13	79.27	81.65	81.08	84.11	88.56
	SP	**99.99**	99.94	98.93	98.26	98.13	97.90	98.17	98.34	99.10
	ACC	96.90	95.32	95.60	97.24	96.93	96.87	97.08	97.43	**98.43**
	PC	51.24	26.92	40.10	65.36	62.10	62.33	63.8	67.51	**78.20**
	CC	70.38	49.96	56.80	77.63	75.03	75.28	76.41	79.31	**86.93**
NT_113953.1	SN	22.80	57.32	29.79	74.05	60.20	64.80	70.53	75.65	**82.74**
	SP	**100.0**	99.74	99.56	98.83	99.27	99.23	99.22	99.13	99.47
	ACC	97.76	98.51	97.53	98.11	98.13	98.23	98.38	98.45	**98.99**
	PC	22.80	52.74	25.96	53.23	48.39	51.59	55.91	58.57	**70.39**
	CC	47.21	69.89	43.61	68.64	64.50	67.25	70.9	73.1	**82.09**
NT_113954.1	SN	31.24	29.86	52.01	76.31	56.92	63.58	70.54	77.68	**78.02**
	SP	**100.0**	99.46	98.72	97.62	98.40	98.13	98.34	98.23	98.23
	ACC	97.47	96.90	97.00	96.83	96.87	96.86	97.32	**97.48**	**97.48**
	PC	31.24	26.19	38.94	47.05	40.12	42.74	49.22	53.15	**53.34**
	CC	55.17	43.81	54.68	63.29	55.65	58.36	64.72	68.53	**68.72**
NT_028395.3	SN	27.11	44.89	54.18	76.68	68.97	72.79	72.52	77.02	**81.52**
	SP	**100.0**	99.47	99.45	98.93	99.27	98.99	99.18	98.9	99.24
	ACC	97.98	97.53	98.19	98.14	98.19	98.06	98.24	98.12	**98.60**
	PC	27.10	39.26	45.36	59.36	57.49	57.17	59.36	59.25	**67.53**
	CC	51.51	57.21	62.26	73.57	72.21	71.75	73.61	73.48	**79.90**

The bold type indicates the best value in all methods.

SN = Sensitivity, SP = Specificity, ACC = Accuracy, PC = Performance coefficient, CC = Correlation coefficient

**Fig 2:**
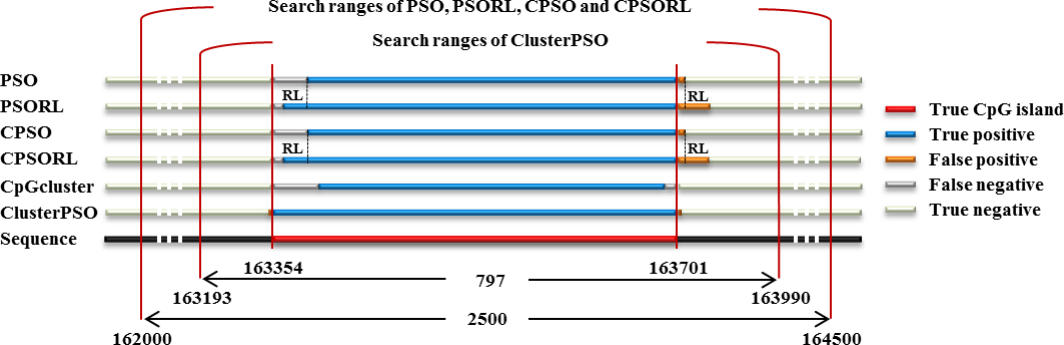
Results of the position of the true CpG island and the positions of the detected CpG islands using the PSO-based methods and ClusterPSO. The search regions for PSO-based methods are also shown to illustrate the difficulty in finding the optimal CpG island. True positive, false positive, false negative and true negative outcomes are clearly shown for comparison between the six methods.

Fig 2 provides a visual illustration of the performance of the six PSO-based methods in contig NT_113952.1. The true CpG island indicates the known CpG island in the NCBI. The true positive indicates the CpG length detected by other PSO-based methods that equal the true CpG island, the false positive indicates the CpG length detected by other PSO-based methods that does not equal the to the true CpG island length. In addition, the false negative indicates the CpG length is not detected by other PSO-based methods but is covered by the true CpG island length, and the true negative indicates the CpG length is not recognized by other PSO-based methods or the true CpG island. According to the experimental results (Fig 2), clusterPSO showed a higher degree of sensitivity and specificity compared to other methods. (clusterPSO: sensitivity (SN) = 93.53, specificity (SP) = 99.63; CpGcluster: SN = 44.45, SP = 70.76; CPSORL: SN = 90.29, SP:99.42; CPSO: SN = 91.43, SP:99.47; PSO: SN = 91.00, SP = 99.47; PSORL: SN = 89.86, SP = 99.43).

Table A in S1 File shows the detailed results of CpG island detection in the six contig sequences by CPSORL and ClusterPSO. The columns GC% (average) and CpG island length show that both CPSORL and ClusterPSO can accurately detect CpG islands as defined by GGF. This table indicates that the ClusterPSO outperforms CPSORL in terms of detecting true CpG islands.

### CpG island detection of human chromosomes 21 and 22

Table 2 shows the CpG island detection results for the whole chromosome sequences using CpGPlot, CpGcluster, CpGProD, CpGIS, PSO, PSORL, CPSO, CPSORL, and ClusterPSO. In chromosome 21, the total length of CpG islands is 1,719,555 bps and the known CpG island coverage is 3.66% in NCBI. ClusterPSO finds the CpG island length is 1,728,357 bps, and the coverage of these CpG islands is 3.68%. In chromosome 22, the total length of CpG islands is 3,114,716 bps and the known CpG island coverage is 6.27% in NCBI. ClusterPSO finds a CpG island length of 3,090,231 bps and coverage of 6.22%. The overall CpG island length and coverage values with known values in NCBI indicate that ClusterPSO outperforms the other methods in terms of detection accuracy. Table 2 provides detailed detection results, including the average length, the minimum length, the maximum length, the GC content, and the O/E ratio for the detected CpG islands. All methods can be applied to detect the GGF-defined CpG islands, but the detection results obtained by CpGcluster for chromosomes 21 and 22 showed a minimum length of 8 bps.

**Table 2:** Comparison of the number of CpG islands identified in the human genome with different methods (NCBI.36).

**Chromosome 21**									
**Chromosome Length (bp): 46,944,329**		Total length of CpG islands(bp): 1,719,555*					CpG island coverage (%): 3.66*		
**Methods**	**CpGPlot**	**CpGcluster**	**CpGProD**	**CpGIS**	**PSO**	**PSORL**	**CPSO**	**CPSORL**	**ClusterPSO**
**Total length of CpG islands**	347,334	639,161	1,072,192	1,280,505	1,440,953	1,564,596	1,527,114	1,607,472	1,728,357
**Number of islands detected**	973	2,703	1,091	3,704	2,648	2,648	2,813	2,813	3,864
**Island coverage (%)**	0.73	1.36	2.28	2.73	3.07	3.3	3.36	3.4	3.68
**Island length (bp)**									
**Average**	357	237	983	346	542	591	561	571	447
**Minimum**	101	8	500	200	202	202	202	202	201
**Maximum**	3,047	3,028	6,732	1,948	4,009	4,020	4,032	4,035	5,785
**GC-content ± SD (%)**	62.17±0.07	65.49±0.07	54.49±0.06	57.98±0.04	54.63±0.05	53.73±0.05	54.12±0.05	53.72±0.05	53.81±0.07
**CpG island O/E ratio ± SD**	0.84±0.1	0.87±0.3	0.63±0.1	0.68±0.1	0.71±0.14	0.64±0.08	0.68±0.11	0.65±0.08	0.68±0.05
**Chromosome 22**									
**Chromosome Length (bp): 49,691,432**	Total length of CpG islands(bp): 3,114,716*						CpG island coverage (%): 6.27*		
**Methods**	**CpGPlot**	**CpGcluster**	**CpGProD**	**CpGIS**	**PSO**	**PSORL**	**CPSO**	**CPSORL**	**ClusterPSO**
**Total length of CpG islands**	679,803	522,748	2,067,653	2,842,255	2,772,787	2,802,675	2,873,255	2,907,983	3,090,231
**Number of islands detected**	1,642	2,186	1,903	6,875	4,571	4,571	4,882	4,882	6,624
**Island coverage (%)**	1.36	1.05	4.16	5.71	5.34	5.64	5.60	5.85	6.22
**Island length (bp)**									
**Average**	414	239	1,087	413	581	613	570	596	467
**Minimum**	200	8	500	200	201	198	201	202	201
**Maximum**	7,902	7,774	8,363	3,339	4,064	4,076	4,064	4,076	5,785
**GC-content ± SD (%)**	63.70±0.08	70.23±0.08	55.84±0.07	55.12±0.06	54.91±0.05	54.50±0.07	55.16±0.05	54.46±0.07	54.91±0.07
**CpG island O/E ratio ± SD**	0.84±0.1	0.95±0.3	0.62±0.1	0.68±0.1	0.66±0.08	0.63±0.05	0.66±0.10	0.63±0.05	0.66±0.05

*The values related to CpG island are obtained from NCBI.

### CpG island detection of entire human genome

Table 3 summarizes the CpG island detection results using CpGcluster, CpGIS, CPSORL, and ClusterPSO in the entire human genome, and shows the statistical values for their detection abilities. ClusterPSO detects 254,783 CpG islands, which is 1.28 times that detected by CpGcluster (198,702), 6.75 times CpGIS (37,729), and 1.22 times CpGIS (208,536). Using ClusterPSO, the average island length is longer than that of CpGcluster (494 bps *vs*. 273 bps). The length distribution of the results of the CpG islands is shown in Figure A in S1 File. The minimum lengths of the various methods are as follows: CpGcluster (8 bps), CpGIS (500 bps), CPSORL (100 bps), and ClusterPSO (200 bps). ClusterPSO determined the greatest CpG island length in a range of 200~749 bps. Figure B in S1 File shows the distributions of the results of GC content and O/E ratios for the CpG islands in 24 chromosomes using ClusterPSO. Most CpG islands have a GC content between 0.5 and 0.7 and an O/E ratio between 0.6 and 1.0, indicating the CpG islands detected using ClusterPSO conform to the GGF criteria. We examined the promoter and transcription start site (TSS) overlap in the CpG island region. A promoter region is defined as −1,500 to +500 bps around the TSS. The TSS number of CPSORL is higher (below 9.33%) than that of CpGcluster and ClusterPSO; the promoter region numbers are also higher (below 11.73%). The TSS numbers and promoter region numbers of CpGcluster are similar to those of ClusterPSO.

**Table 3:** Comparison of four methods for CpG island detection in the entire human genome.

Methods	CpGcluster	CpGIS	CPSORL	ClusterPSO
**Genome length**	2.86 × 10^9^			
**Number of predicted island**	198,702	37,729	208,536	254,783
**Coverage (%)**	1.90	1.44	4.1	4.27
**Island length**				
**Average**	273 ± 246	1,090±717	572±469	494±572
**GC content ± SD**	63.78±7.50	60.64±5.06	53.90±5.25	53.76±4.80
**O/E ratio ± SD**	0.855±0.265	0.717±0.082	0.649±0.087	0.678±0.102
**TSSs**	21,741	15,106	25,477	23,757
**Promoter regions**	29,156	13,196	54,356	29,880

Table B in S1 File compares the performance of CPSORL and ClusterPSO in 24 chromosomes of the human genome. ClusterPSO is found to outperform CPSORL in terms of the average values for sensitivity, *SP*, accuracy, *PC*, and *CC*, which clearly indicates that ClusterPSO provides significantly improved accuracy in CpG island detection. For the Wilcoxon Signed-Rank test, the *p*-values of sensitivity, specificity, accuracy, *PC*, and *CC* are 1.82E-05, 0.807, 1.81E-05, 1.82E-05, and 1.82E-05 in 24 chromosomes, respectively. The statistical analysis indicates that specificity values are similarly high for both CPSORL and ClusterPSO (98.97 *vs*. 98.99). However, the other *p*-values indicate the strong superiority of ClusterPSO over CPSORL for CpG island detection in the human genome.

### Detection time comparison for the PSO-based methods and ClusterPSO

**Fig 3:**
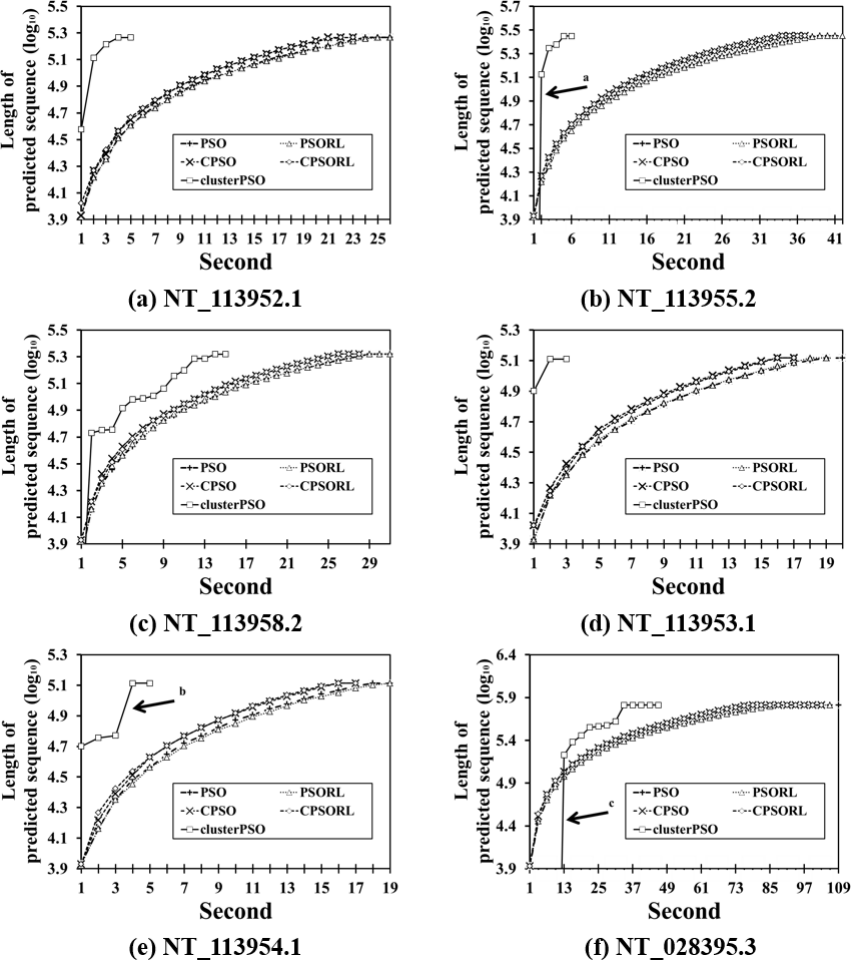
Comparison of CPU time and search efficiency amongst six methods for the six contig sequences. The CPU times for PSO, CPSO, PSORL, CPSORL and ClusterPSO are shown to assess relative search efficiency in the six contig sequences. The horizontal axis represents the implementation time, and the vertical axis represents the log_10_ value for the presently-detected position in the sequence. Arrows a and c show that the CpGcluster step handles long sequences, hence sequence scanning may proceed more slowly at first than in other methods. Arrows a, b and c show that the CpGcluster step detects very few CpG island candidates in the region.

Fig 3 shows the computation times of four PSO-based methods and ClusterPSO in six contig sequences. The PSO, PSORL, CPSO, and CPSORL show similar implementation times, but the ClusterPSO shows excellent results in terms of reducing implementation times. The arrow in Fig 3C shows the algorithm implementation stops near a value of 4.9, but other methods stop near 5.1. The arrow in Fig 3E shows that the rate of detection for ClusterPSO increases from location 4.8 to location 5.1 in the detection sequence. The region in NT_113954.1 does not detect any CpG islands from the CpGcluster step, and the known solution according to NCBI also indicates the regions include very few CpG islands. Thus, ClusterPSO can effectively reduce the implementation time by eliminating unnecessary regions in the sequence. Fig 3B and 3F show that CpGcluster can handle long sequences quickly, where time spent is indicated by the arrow in the figures. Although sequence scanning may proceed more slowly at first than in other methods, it provides a strong time advantage for complete genome searches.

## Discussion

### ClusterPSO method

We propose a procedure (ClusterPSO) for CpG island detection to overcome the drawbacks of CpGcluster and reduce the search difficulty of PSO. Although gene analysis targets a whole mammalian genome, CpG island lengths are typically 300–3,000 bps in genomes, and PSO methods proceed piece-by-piece. Fig 2 illustrates the advantages of ClusterPSO over other methods by comparing the search results of six methods in contig NT_113952.1. A true CpG island is located in region of 163,354 to 163,701 bps (length: 347 bps). Of the six methods, CpGcluster detects the shortest CpG island (region: 163,393–163,690, length: 297 bps). In this case, CpGcluster produces the fewest false positives, but has the lowest sensitivity (85.59%, 297/347) due to its short length. Fig 2 shows the search region for all PSO-based methods. Chuang *et al*. proposed several improved PSO methods for detecting CpG islands by dividing the whole sequence into many small sequences, with regions defined as being 2,500 bps in length [10]. The possible search space is 2,500 bps, and the resulting huge number of possible solutions makes it difficult for PSO-based methods to accurately detect the optimal CpG island position. Although reinforcement learning (RL) can improve PSO search ability in terms of true positives, it might also result in increased false positives due to the extension of the sequence. Based on the own-relax criteria of CpGcluster, ClusterPSO extends the search region to predict optimal CpG islands. The extended search region successfully reduces the number of pairwise intersections to 797, and the true CpG island is included in the search region. Fig 2 shows that ClusterPSO provides the highest true positive detection and the lowest rate of false positives. The analysis below explains how combining CpGcluster and PSO can mitigate the individual disadvantages of each method.

In the Hackenberg et al. study, three thresholds (25^th^, 50^th^ and 75^th^) are used to test CpGcluster for CpG island detections. Hackenberg et al. suggested using the region between 50^th^ and 75^th^ to detect CpG islands. Raising the distance threshold to the 75^th^ percentile obtains longer islands, and can thus increase the sensitivity by more than 20% while only minimally improving overall accuracy [11]. Lowering the *p*-value threshold beyond 10^−5^ slightly increased *SP* but also clearly decreased *SN*, thus lowering overall global accuracy. In this study, ClusterPSO selected the 65^th^ to balance the *SN* and *SP*. The PSO step can filter CpG islands again, indicating the number of non-CpG island regions can be reduced. When the *p*-value is small, the computational time of ClusterPSO can be increased due to the large number of detected CpG island candidates. Therefore, we suggest the ClusterPSO use the 65^th^ to detect CpG islands, and the region of *p*-value thresholds is suggested between the 50^th^ and 75^th^ to detect CpG islands.

### Comparison of the true positive and false positive rates for CpGcluster, PSO-based methods, and ClusterPSO

Our previous study proposed PSO-based methods to detect the CpG islands with high true positive rates (*TPR*) and low false positive rates (*FPR*) [10]. These PSO-based methods reveal slightly higher *FPR* than CpGcluster, but also show substantially higher *TPR* than CpGcluster and other methods. Overall, ClusterPSO is obviously superior to all other PSO-based methods. Figure C in S1 File shows the XY charts for comparing the *TPR* and *FPR* of CpGcluster, PSO-based methods, and ClusterPSO. The upper-left location of the chart indicates the better CpG island detection results, and the figure indicates that ClusterPSO outperforms the other five methods in terms of *TPR*, but has a slight disadvantage in terms of *FPR*. In addition, this figure indicates that the disadvantages of both CpGcluster and PSO can be greatly improved by combining the two methods, making our approach highly effective for CpG island detection.

### Search stability comparison for all of the PSO-based methods

Search stability is an important performance criterion for PSO-based methods. Different random seeds have an impact on CpG island detection. Figure D in S1 File shows a box plot which compares the relative stabilities of sensitivity, specificity, accuracy, *PC*, and *CC* for PSO-based, and ClusterPSO through 1,000 test iterations. Given an identical random seed, all PSO-based methods have the same random value in each generation, and some seed values may result in search failure, thus reducing the accuracy in CpG island detection. However, the ClusterPSO still detects CpG islands with a high degree of accuracy, even with unfavourable seed values (the error bars near the left boxes indicate 10^th^ percentiles). In the box plot, a small box indicates better stability in each performance measurement. In terms of stability, ClusterPSO outperforms the other PSO-based methods.

### ClusterPSO performance for the entire human genome

In entire human genome analysis, ClusterPSO performs better than CPSORL in terms of sensitivity, specificity, accuracy, *PC*, and *CC* values (Table B in S1 File), and CpG island prediction accuracy and coverage rate. Table C in S1 File shows that ClusterPSO outperforms PSO-based methods and CpGcluster in terms of detecting overlapping CpG islands, indicating that ClusterPSO can correctly detect CpG islands.

The short lengths of CpG islands may result in high O/E ratio and high GC content levels, but also results in low sensitivity [13]. However, ClusterPSO is based on CpGcluster and uses the CpG criteria to detect CpG islands, thus the O/E ratio and GC content of ClusterPSO are smaller than those of CpGcluster, but with substantially improved sensitivity. Figure E in S1 File shows the box plot of the O/E ratio for each interval length in 24 chromosomes. The figures show the O/E ratios for all boxes remain between 0.6 and 0.7 in each interval length, and the box width indicates which box corresponds to the O/E value for CpG islands in the 25^th^ and 75^th^ percentiles of a given CpG island class. This indicates that CpG islands detected by ClusterPSO can follow CpG island criteria without being impacted by CpG island length.

Hackenberg *et al*. compared CpGcluster against the sliding window method. They found that CpGcluster co-localized more specifically to TSSs, and many of the small CpG islands detected by CpGcluster may be functional, given the overlap with conserved elements or promoter regions [12]. The results for TSSs and the promoter regions (Table 3) show that ClusterPSO inherits these advantages from CpGcluster because ClusterPSO is based on CpGcluster detection.

The Hackenberg *et al*. study found that small CpG clusters (length < 200bps) may have important biological implications [11]. In our study, ClusterPSO used the GGF to define the CpG islands (length > 200 bps, GC content > 50%, and O/E > 0.6), in which the minimum length defined in the particle (see Stage 2) may limit the predictive potential of ClusterPSO. This minimum length limitation can be eliminated for the detection of additional CpG islands with important biological meanings but the prediction results may reduce the prediction sensitivity due to the great number of small CpG islands.

This study combines CpGcluster and PSO methods to design a simple and accurate ClusterPSO method to detect CpG islands in human DNA sequences. This combination has several advantages over CpGcluster and PSO alone: (1) the implementation time and search stability of PSO can be significantly improved by pre-treatment with CpGcluster; (2) the short CpG island length of CpGcluster may not meet CpG island criteria, but it can be improved by an accurate PSO prediction; (3) ClusterPSO only requires six parameters which is easy to implement; and (4) future improvements to CpGcluster and PSO will greatly enhance the accuracy of ClusterPSO detection.

## Supporting Information

S1 File Additional results and imputation commands. The supplementary file includes the computational details of ClusterPSO and supplementary Figures and Tables. Length distribution of the results of CpGIS, CpGCluster, CPSORL, and ClusterPSO in the human genome **(Figure A)**. Distribution of the results of CpG islands in the human genome (**Figure B)**. XY charts comparing the true positive and false positive rates amongst the six methods for six contig sequences **(Figure C)**. Box plot comparing the stability of five methods in six contig sequences **(Figure D)**. Box plot of the O/E ratio for each interval length in the human genome **(Figure E)**. Number of CpG islands located in gene regions identified with CPSORL and ClusterPSO **(Table A)**. Performance measurement of ClusterPSO and CPSORL for all chromosomes in the human genome **(Table B)**. Number of detection CpG islands overlapping on true CpG islands for CpGcluster, CPSORL and ClusterPSO for all chromosomes in the human genome **(Table C)**.
(DOC)
