# Supplementary material for: A Hybrid Approach for CpG Island Detection in the Human Genome
Source: PLoS One. 2016 Jan 4;11(1):e0144748. doi: 10.1371/journal.pone.0144748 (PMC4705099; doi:10.1371/journal.pone.0144748)
Supplement: S1 File — The supplementary file includes the computational details of ClusterPSO and supplementary Figures and Tables. Length distribution of the results of CpGIS, CpGCluster, CPSORL, and ClusterPSO in the human genome (Figure A). Distribution of the results of CpG islands in the human genome (Figure B). XY charts comparing the true positive and false positive rates amongst the six methods for six contig sequences (Figure C). Box plot comparing the stability of five methods in six contig sequences (Figure D). Box plot of the O/E ratio for each interval length in the human genome (Figure E). Number of CpG islands located in gene regions identified with CPSORL and ClusterPSO (Table A). Performance measurement of ClusterPSO and CPSORL for all chromosomes in the human genome (Table B). Number of detection CpG islands overlapping on true CpG islands for CpGcluster, CPSORL and ClusterPSO for all chromosomes in the human genome (Table C). (DOC) [file pone.0144748.s001.doc]

*Additional file —A hybrid approach for CpG island detection in the human genome*

# A hybrid approach for CpG island detection in the human genome

Cheng-Hong Yang^1^, Yu-Da Lin^1^, Yi-Cheng Chiang^1^, Li-Yeh Chuang^2^,*

^1^ Department of Electronic Engineering, National Kaohsiung University of Applied Sciences, Kaohsiung, Taiwan.

^2^ Department of Chemical Engineering & Institute of Biotechnology and Chemical Engineering, I-Shou University, Kaohsiung, Taiwan.

The supplementary file shows the computational details of ClusterPSO. The parameters for CpGcluster are set as follows: *percentile_limit* 65% and *p*-*value_limit* 0.01.

**CpGcluster step:**

**Step 1:** Record the CpGs position in the input sequence.

| CpG | CpG_1_ | CpG_2_ | CpG_3_ | CpG_4_ | CpG_5_ | … | CpG_1694_ | CpG_1695_ | CpG_1696_ | CpG_1697_ | CpG_1698_ |
| --- | --- | --- | --- | --- | --- | --- | --- | --- | --- | --- | --- |
| location | 358 | 590 | 905 | 1182 | 1309 | … | 183983 | 183999 | 184235 | 184239 | 184256 |

**Step 2:** Calculate the distance between adjacent CpGs..

| distance | 233 | 316 | 278 | 128 | 306 | … | 17 | 237 | 5 | 18 |  |
| --- | --- | --- | --- | --- | --- | --- | --- | --- | --- | --- | --- |

**Step 3:** Sort distance values obtained in the step 2.

| Sort_array | 3 | 3 | 3 | 3 | 3 | … | 1202 | 1250 | 1462 | 2016 |  |
| --- | --- | --- | --- | --- | --- | --- | --- | --- | --- | --- | --- |

**Step 4:** Set the largest distance (*dist_threshold*) between the adjacent clusters.

*dist_threshold* = Sort [*CpG_num ** *percentile_limit*] = 79

**Step 5:** If the distance between the adjacent CG dinucleotide smaller than *dist_threshod*, combine the adjacent CG dinucleotides as a cluster.


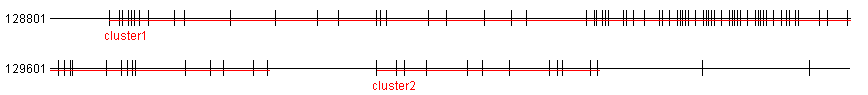


cluster_1_ (128859, 129819);

cluster_2_ (129927, 130149);

**Step 6:** Calculate the *p*-value of all clusters.

$$P_{N,p}^{cum}(x<=n_{f})=\sum_{x=0}^{n_{f}} \left(\begin{matrix} x-(N+1)-1 \\ (N-1)-1 \end{matrix}\right)\times p^{N-1}\times(1-p)^{x}$$

$$n_{f}=L-2\times N$$

$$p=\frac{N_{s}}{N_{is}}$$

*N_s_* = *CG_num* = 1698;

*N_is_* = *sequence_length* – (*CG_num* + 1) = 184355 –1698 = 182657;

*p* = 0.0092961123855095;

*p*-value (cluster_1_) = 1.9137580193525646E–51;

**Step 7:** Executing PSO for CpG clusters with sufficient *p-values*.


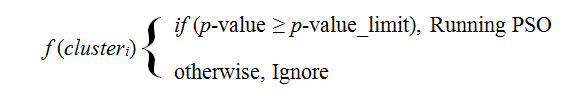


**PSO step:**

We example the cluster_1_ (128859, 129819) to running PSO;

Using cluster_1_ (128859, 129819) as an example, with PSO parameters as follows: number of particles is 300, number of generations is 100, *r_1_* (*rand* (0, 1)), *r_2_* (*rand* (0, 1)), *v_max* is 4, and *v_min* is –4.

**Step 1:** Define the search range, *range_min* = 128659; *range_max* = 130019.

**Step 2:** Randomly generate initial particle populations.

*Particle_i_* = (*F_s_*, *F_l_*) and *Velocity_i_* = (*v_1_*, *v_2_*);
*Fs_max* = 130019 – 128659 – 200 = 1360; *Fs_min* = 0;

*Fl_max* = 130019 – 128659 – *F_s_* = 1360 – *F_s_*; *Fl_min* = 200

Initial particle swarm :

*Particle_1_* = (848, 234); *Velocity_1_* = (-3.43, 2.15);

*Particle_2_* = (263, 884); *Velocity_2_* = (1.28, -3.76);

*Particle_3_* = (940, 264); *Velocity_3_* = (3.56, 3.28);

*Particle_4_* = (1159, 201); *Velocity_4_* = (0.32, -2.73);

*Particle_5_* = (636, 321); *Velocity_5_* = (-3.25, -0.7);

**Step 3:** Calculating the fitness value of the particles.

$$GC(P_{i})=\frac{\#C+\#G}{\#A+\#T+\#C+\#G}$$

$$\text{Obs}_{\text{CpG}}\text{/Exp}_{\text{CpG}}\text{ }(P_{i})=\frac{\frac{\#CpG}{CpG_{length}}}{\frac{\#C}{CpG_{length}}\times\frac{\#G}{CpG_{length}}}$$

$$CpG_{len} (P_{i})= \frac{(\#A+\#T+\#C+\#G)}{F_{s\_max}\text{－}F_{s\_min}}$$

$$Fitness\text{(}P_{i}\text{)}=GC\text{(}P_{i}\text{)}\times0.5+Obs_{CpG}/Exp_{CpG}\text{(}P_{i}\text{)}\times0.25+CpG_{len} \text{(}P_{i}\text{)}\times0.25$$

| *Particle_1_* | *C_num*: 75; *G_num*: 77; *CpG_num*: 23; *length*: 234  *fitness_1_* = 0.6496$\times$0.5 + 0.9319$\times$0.25 + 0.1721$\times$0.25 = 0.6008; |
| --- | --- |
| *Particle_2_* | *C_num*: 354; *G_num*: 251; *CpG_num*: 72; *length*: 884  *fitness_2_* = 0.6844$\times$0.5 + 0.7163$\times$0.25 + 0.65$\times$0.25 = 0.6838; |
| *Particle_3_* | *C_num*: 68; *G_num*: 92; *CpG_num*: 14; *length*: 264  *fitness_3_* = 0.6061$\times$0.5 + 0.5908$\times$0.25 + 0.1941$\times$0.25 = 0.4993; |
| *Particle_4_* | *C_num*: 47; *G_num*: 79; *CpG_num*: 5; *length*: 201  *fitness_4_* = 0.6269$\times$0.5 + 0.2707$\times$0.25 + 0.1478$\times$0.25 = 0.418; |
| *Particle_5_* | *C_num*: 149; *G_num*: 93; *CpG_num*: 46; *length*: 321  *fitness_5_* = 0.7539$\times$0.5 + 1.0656$\times$0.25 + 0.236$\times$0.25 = 0.7024; |

**Step 4:** Determine whether each particle complies with the CpG island criteria. ($GC(P_{i})\geq0.5 \text{；Obs}_{\text{CpG}}\text{/Exp}_{\text{CpG}}\text{ }(P_{i})\geq0.6 \text{； }length\geq200$).

*CpGI_1_* = (129507, 129741), *CG%* = 0.6496, *OE%* = 0.9319, *fitness_1_* = 0.6008；

*CpGI_2_* = (128922, 129806), *CG%* = 0.6844, *OE%* = 0.7163, *fitness_2_* = 0.6838；

*CpGI_3_* = (129295, 129616), *CG%* = 0.7539, *OE%* = 1.0656, *fitness_3_* = 0.7024；

If overlap occurs, retain the particle of highest fitness. After overlap process,

*CpGI_1_* = (129295, 129616); *fitness_1_* = 0.7024

**Step 5:** Update *pbest_i_* and *gbest*.

*gbest* = (636, 321); *gbest_fit* = 0.7024;

*pbest_1_* = (848, 234); *pbest_fit_1_* = 0.6008;

*pbest_2_* = (263, 884); *pbest_fit_2_* = 0.6838;

*pbest_3_* = (940, 264); *pbest_fit_3_* = 0.4993;

*pbest_4_* = (1159, 201); *pbest_fit_4_* = 0.418;

*pbest_5_* = (636, 321); *pbest_fit_5_* = 0.7024;

**Step 6:** Update particle

$v{}_{id}^{new}=w\times v_{id}^{old}+c_{1}\times r_{1}\times\left(pbest_{id}-x_{id}^{old}\right)+c_{2}\times r_{2}\times\left(gbest_{d}-x_{id}^{old}\right)$

$$x_{i}^{new}=x_{i}^{oid}+v_{i}^{new}$$

$$w=(w_{\max}-w_{\min})\times\frac{move_{\max}-move_{i}}{move_{\max}}+w_{\min}$$

*Ex: Particle_1_* = (848, 234); *Velocity_1_* = (-3.43, 2.15);

$$\begin{matrix} v_{1}^{new}=-\text{33.19 }=\text{ 0.9 }\times\text{ (}-\text{3.43) }+\text{ 0.73 }\times\text{ 2 }\times\text{ (848 }-\text{ 848) }+\text{ 0.07 }\times\text{ 2 }\times\text{ (636 }-\text{ 848)} \text{；} \\ \text{because}-33.19>-\text{4，hence }v_{1}^{new}=-4 \text{；then} x_{1}^{new}=844=848+(-4) \text{；} \end{matrix}$$

$$\begin{matrix} v_{12}^{new}=\text{135.59 }=\text{ 0.9 }\times\text{ 2.15 }+\text{ 0.07 }\times\text{ 2 }\times\text{ (234 }-\text{ 234) }+\text{ 0.77 }\times\text{ 2 }\times\text{ (321 }-\text{ 234)} \text{；} \\ \text{becausae} \text{135.59 }>\text{ 4} \text{，hence} v_{12}^{new}=4 \text{；then} x_{12}^{new}=238=234+4 \text{；} \end{matrix}$$

*Particle_1_* = (844, 238); *Velocity_1_* = (-4, 4);

*Particle_2_* = (267, 880); *Velocity_2_* = (4, -4);

*Particle_3_* = (936, 268); *Velocity_3_* = (-4, 4);

*Particle_4_* = (1155, 205); *Velocity_4_* = (4, 4);

*Particle_5_* = (633, 320); *Velocity_5_* = (-2.925, -0.63);

**Repeat the third step:**

| *Particle_1_* | *C_num*: 77; *G_num*: 78; *CpG_num*: 24; *length*: 238;  *fitness_1_* = 0.6513$\times$0.5 + 0.951$\times$0.25 + 0.175$\times$0.25 = 0.6071; |
| --- | --- |
| *Particle_2_* | *C_num*: 353; *G_num*: 249; *CpG_num*: 71; *length*: 880;  *fitness_2_* = 0.6841$\times$0.5 + 0.7108$\times$0.25 + 0.6471$\times$0.25 = 0.6815; |
| *Particle_3_* | *C_num*: 71; *G_num*: 93; *CpG_num*: 15; *length*: 268;  *fitness_3_* = 0.6119$\times$0.5 + 0.6088$\times$0.25 + 0.1971$\times$0.25 = 0.5074; |
| *Particle_4_* | *C_num*: 47; *G_num*: 82; *CpG_num*: 5; *length*: 205;  *fitness_4_* = 0.6293$\times$0.5 + 0.266$\times$0.25 + 0.1507$\times$0.25 = 0.4188; |
| *Particle_5_* | *C_num*: 149; *G_num*: 90; *CpG_num*: 46; *length*: 320;  *fitness_5_* = 0.7469$\times$0.5 + 1.0977$\times$0.25 + 0.2353$\times$0.25 = 0.7067; |

**Repeat step 4:**

*CpGI_1_* = (129295, 129616), *fitness_3_* = 0.7024; (Previous generations of winners)

*CpGI_2_* = (129503, 129741), *CG%* = 0.6513, *OE%* = 0.951, *fitness_2_* = 0.6071;

*CpGI_3_* = (128926, 129806), *CG%* = 0.6841, *OE%* = 0.7108, *fitness_3_* = 0.6815;

*CpGI_4_* = (129595, 129863), *CG%* = 0.6119, *OE%* = 0.6088, *fitness_3_* = 0.5074;

*CpGI_5_* = (129292, 129612), *CG%* = 0.7469, *OE%* = 1.0977, *fitness_3_* = 0.7067;

After overlap process, *CpGI_1_* = (129292, 129612); *fitness_1_* = 0.7067

**Repeat step 5:**

*gbest* = (633, 320); *gbest_fit* = 0.7067;

*pbest_1_* = (844, 238); *pbest_fit_1_* = 0.6071;

*pbest_2_* = (267, 880); *pbest_fit_2_* = 0.6815;

*pbest_3_* = (936, 268); *pbest_fit_3_* = 0.5074;

*pbest_4_* = (1155, 205); *pbest_fit_4_* = 0.4188;

*pbest_5_* = (633, 320); *pbest_fit_5_* = 0.7067;

**In step 4 of the last iteration:** the particles meet the criteria CpG island comprises:

*CpGI_1_* = (129344, 129663), *fitness_1_* = 0.7207; (Previous generations of winners)

*CpGI_2_* = (129398, 129717), *CG%* = 0.7147, *OE%* = 1.1143, *fitness_2_* = 0.6946；

*CpGI_3_* = (129236, 130019), *CG%* = 0.6769, *OE%* = 0.7262, *fitness_3_* = 0.6639；

*CpGI_4_* = (129344, 129663), *CG%* = 0.7524, *OE%* = 1.1433, *fitness_4_* = 0.7207；

*CpGI_5_* = (129418, 129737), *CG%* = 0.7022, *OE%* = 1.0850, *fitness_5_* = 0.6810；

*CpGI_6_* = (129312, 129631), *CG%* = 0.7680, *OE%* = 1.0850, *fitness_6_* = 0.7139；

After overlap process, *CpGI_1_* = (129344, 129663), *fitness_1_* = 0.7207;


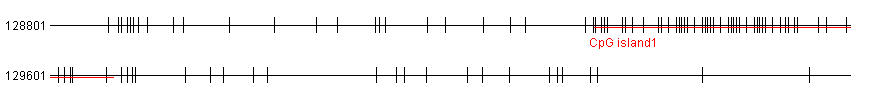


**Figure A.** Length distribution of the results of CpGIS, CpGCluster, CPSORL, and ClusterPSO in the human genome.


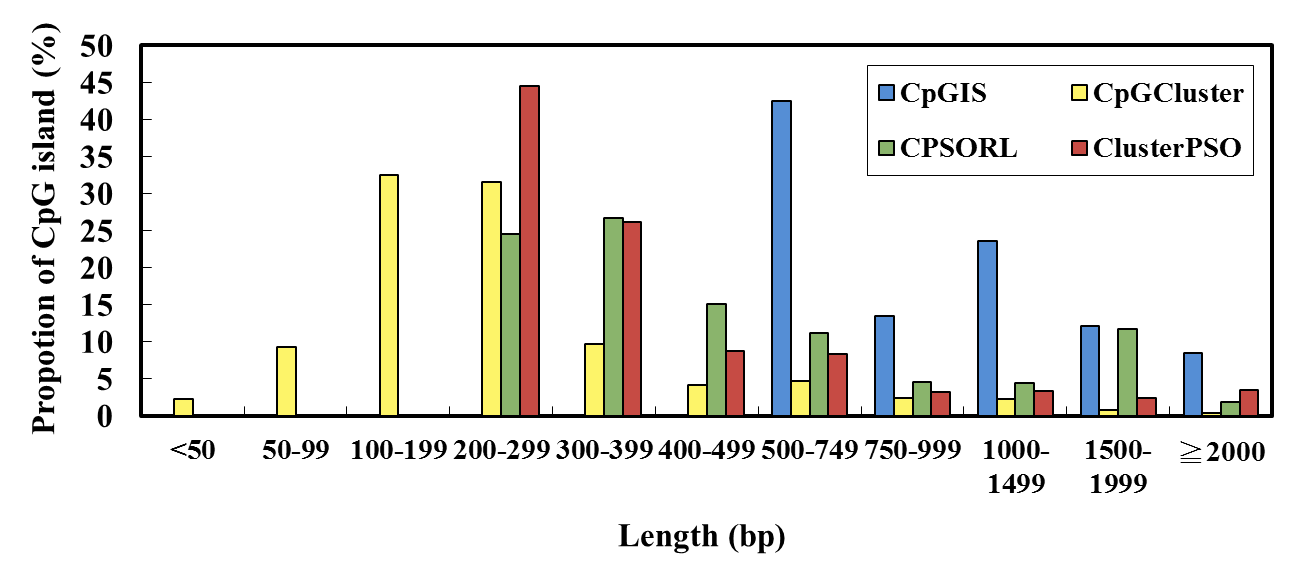


**Figure B.** Distribution of the results of CpG islands in the human genome. The blue dots indicate the CpG islands, and the x and y axes are the GC% and the O/E ratio, respectively. Most CpG islands lie in the region of 50–70% GC, and the O/E ratio lies between 0.6 and 1.0.

| **Chromosome 1**  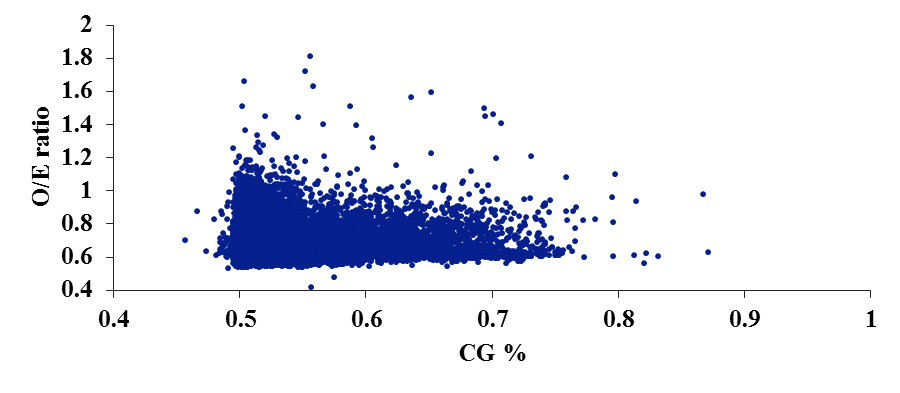 |
| --- |
| **Chromosome 2**  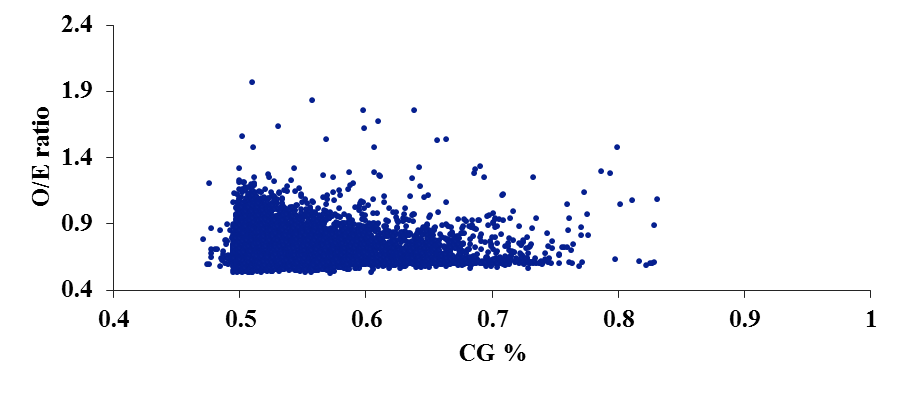 |
| **Chromosome 3**  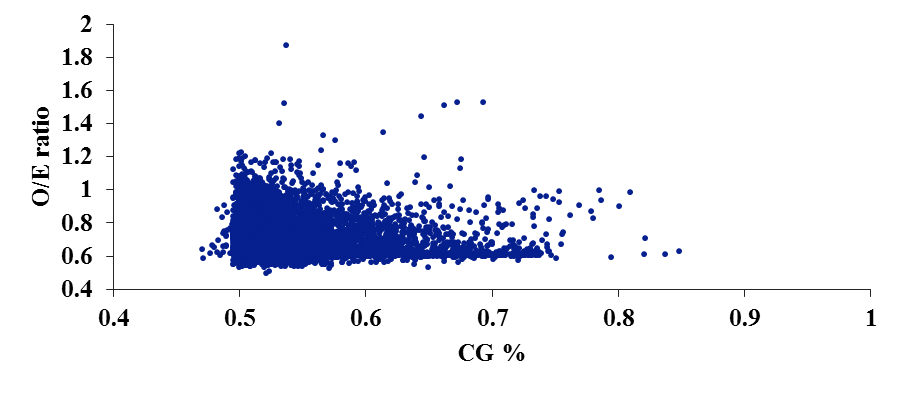 |
| **Chromosome 4**  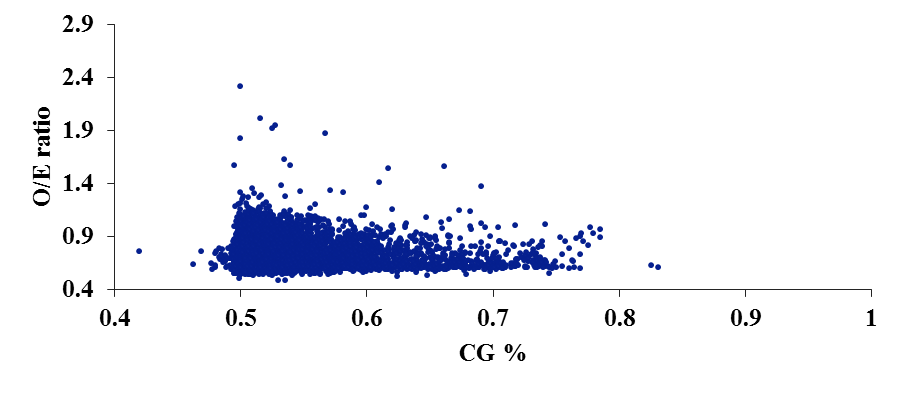 |
| **Chromosome 5**  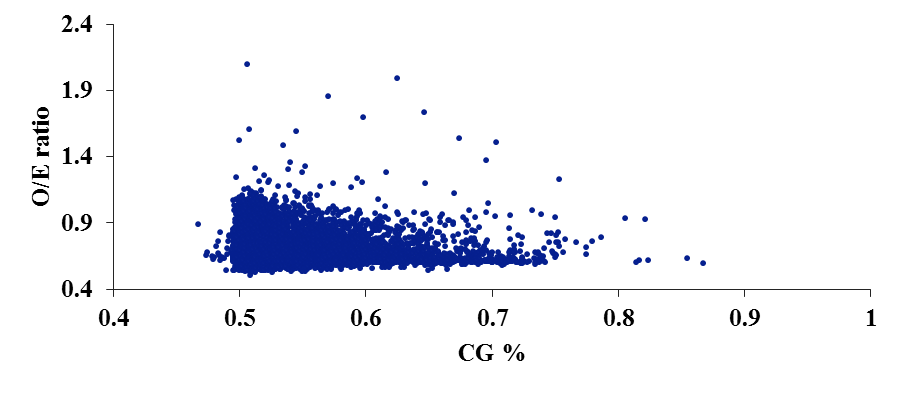 |
| **Chromosome 6**  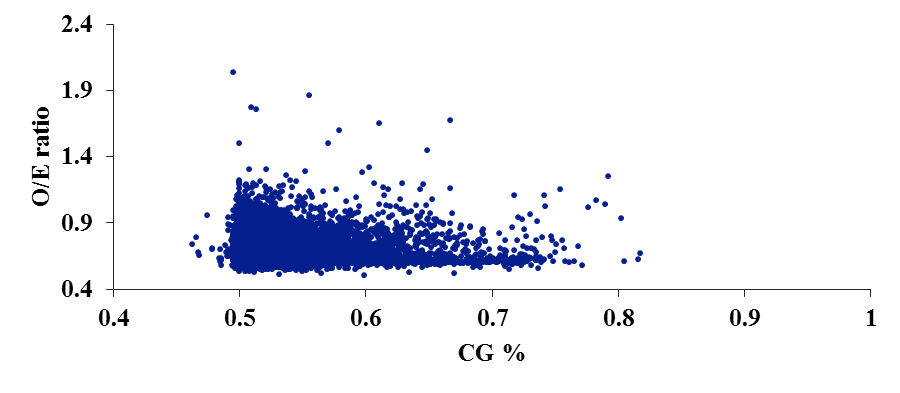 |
| **Chromosome 7**  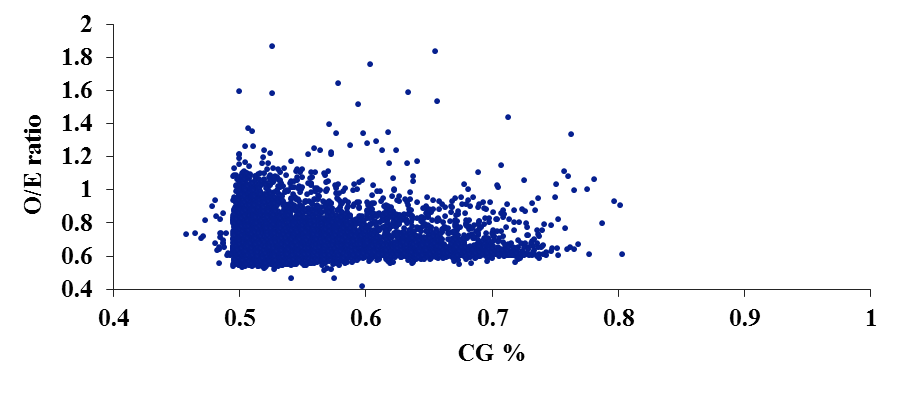 |
| **Chromosome 8**  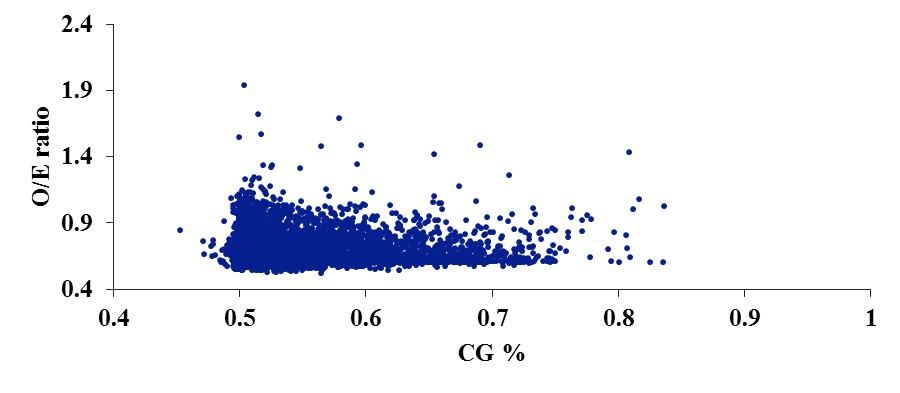 |
| **Chromosome 9**  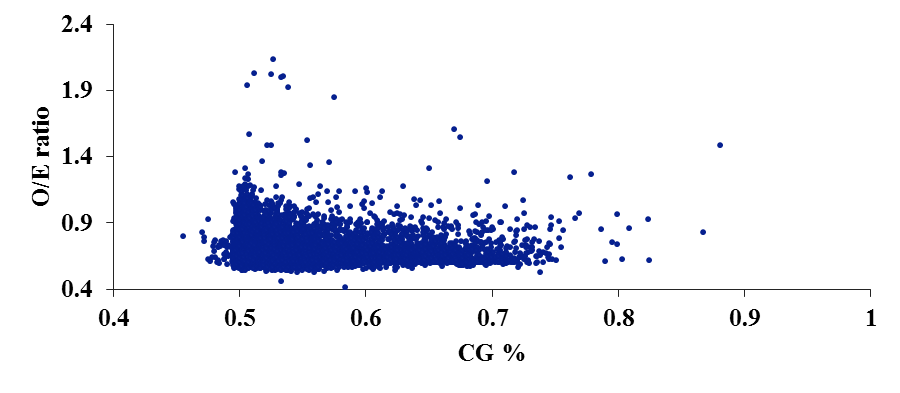 |
| **Chromosome 10**  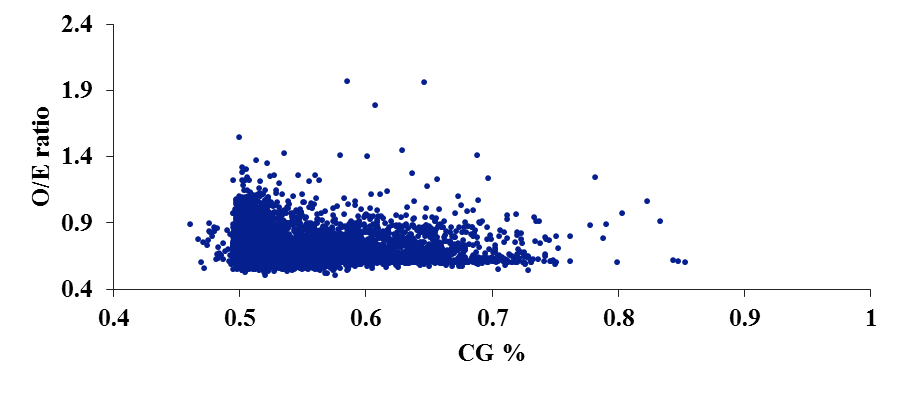 |
| **Chromosome 11**  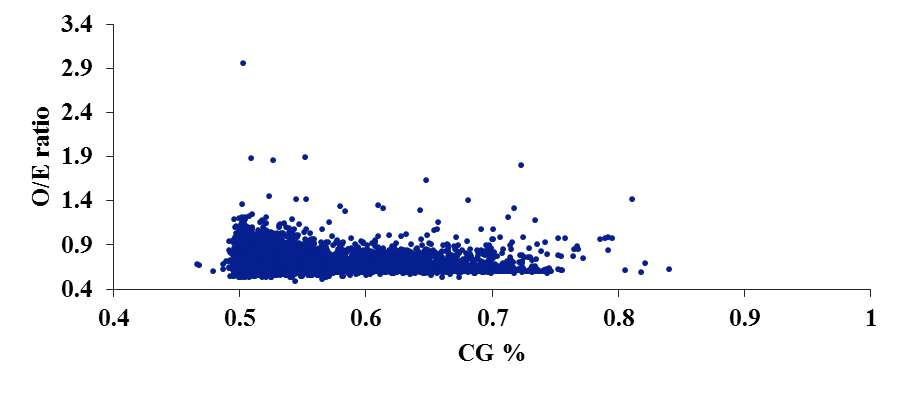 |
| **Chromosome 12**  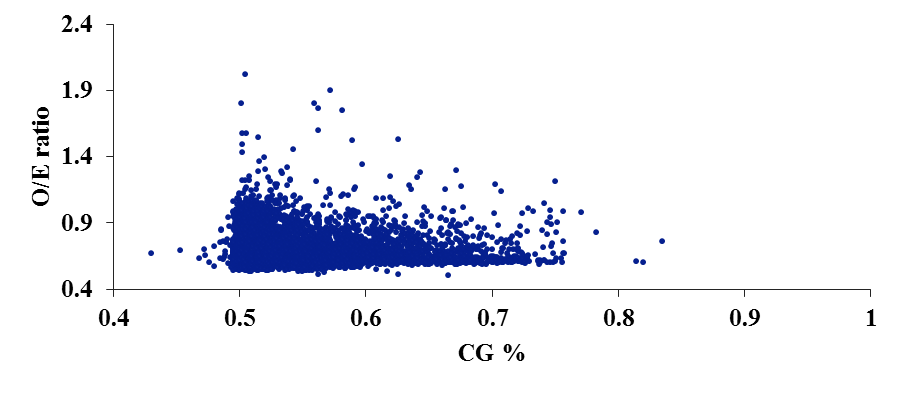 |
| **Chromosome 13**  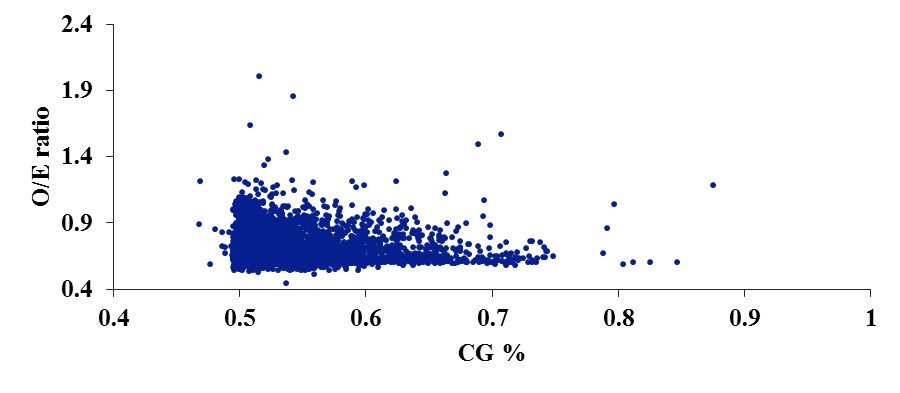 |
| **Chromosome 14**  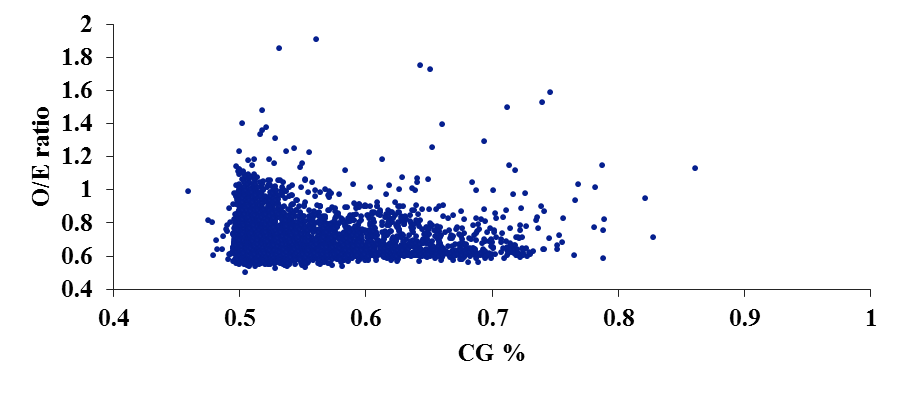 |
| **Chromosome 15**  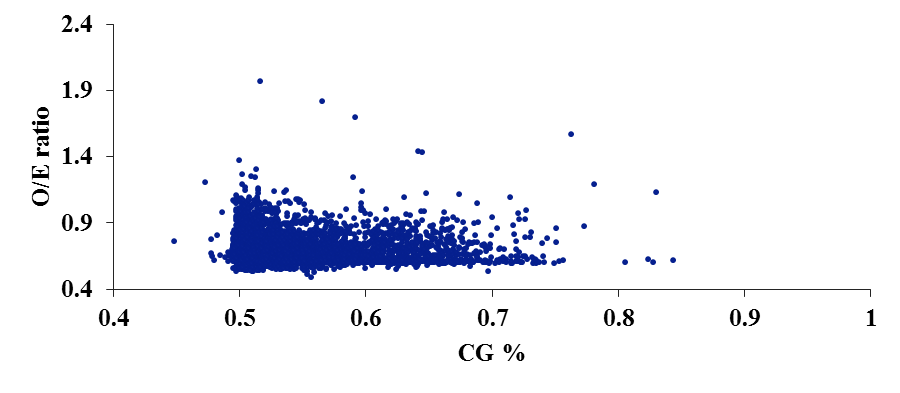 |
| **Chromosome 16**  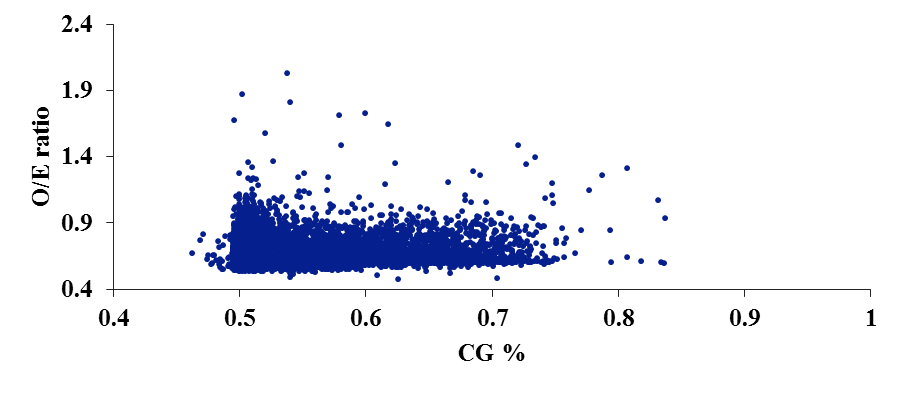 |
| **Chromosome 17**  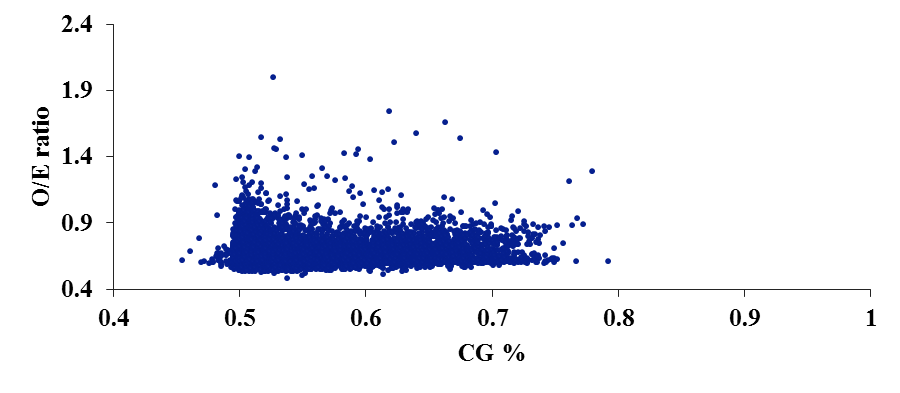 |
| **Chromosome 18**  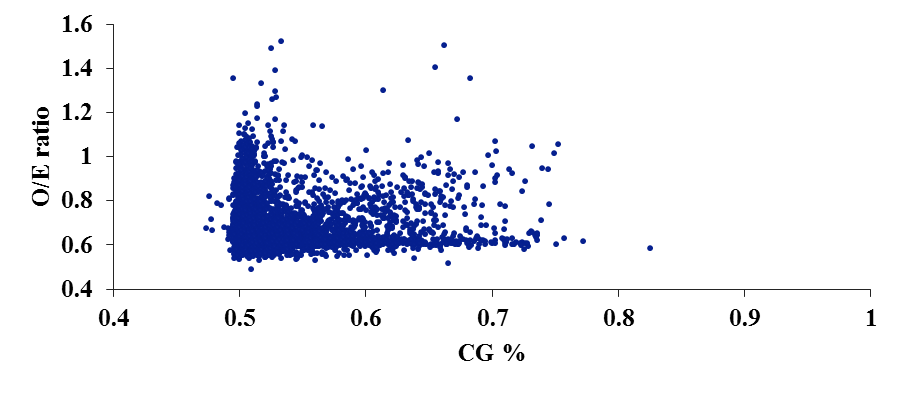 |
| **Chromosome 19**  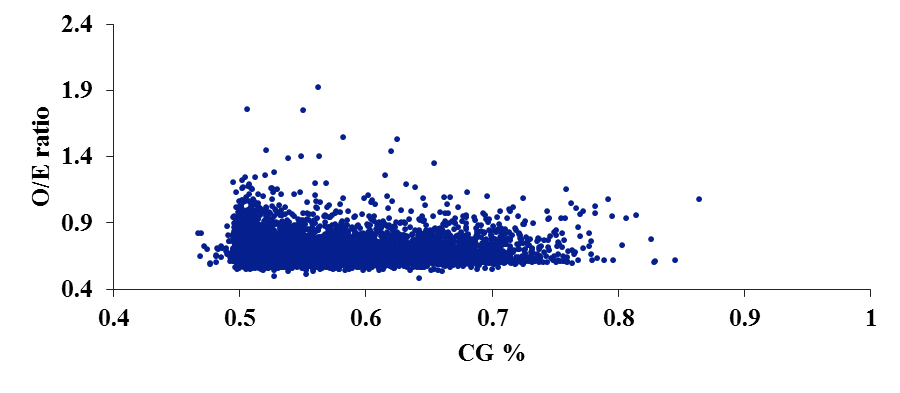 |
| **Chromosome 20**  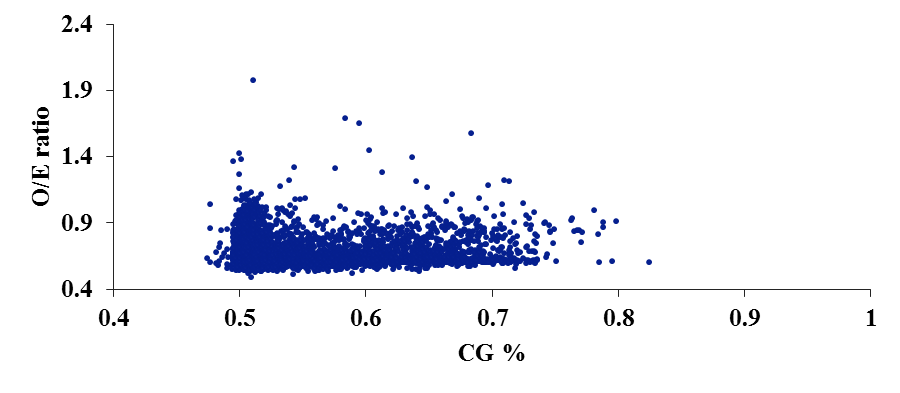 |
| **Chromosome 21**  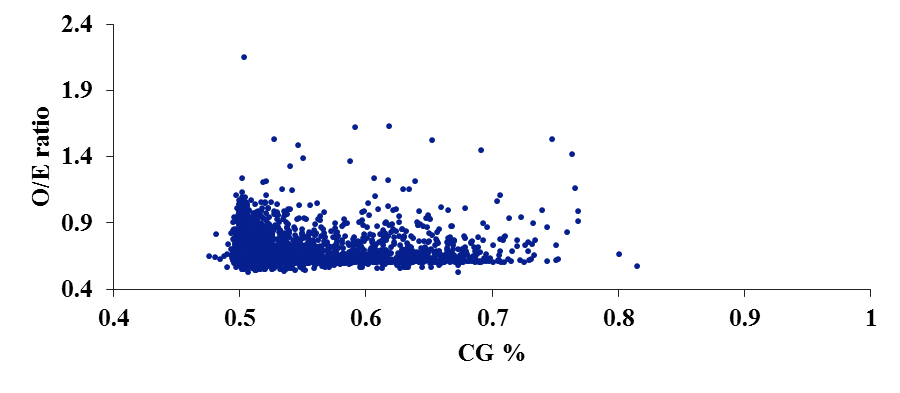 |
| **Chromosome 22**  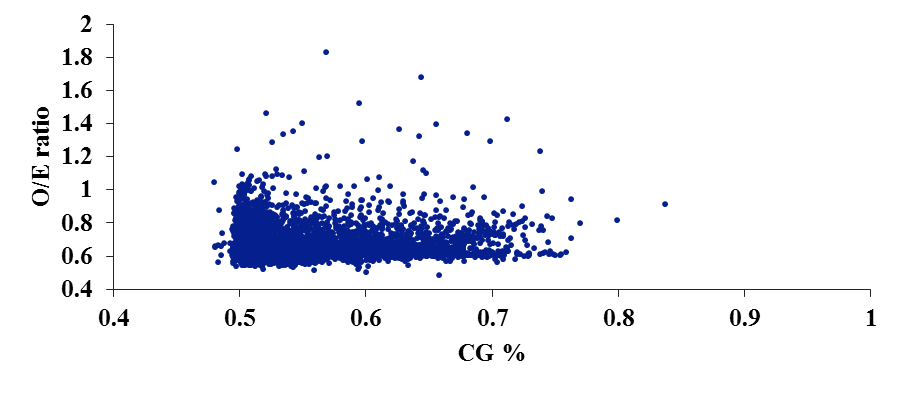 |
| **Chromosome X**  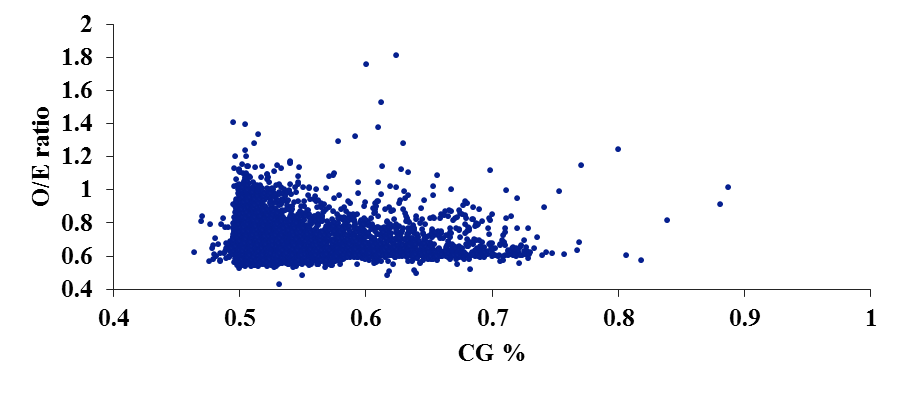 |
| **Chromosome Y**  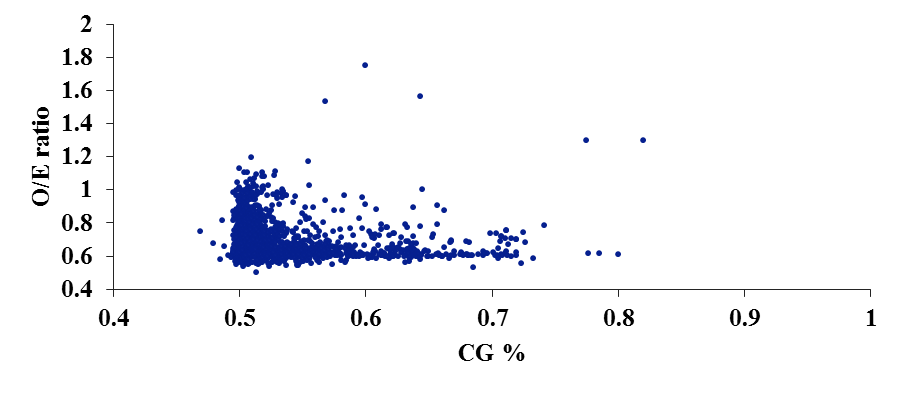 |

**
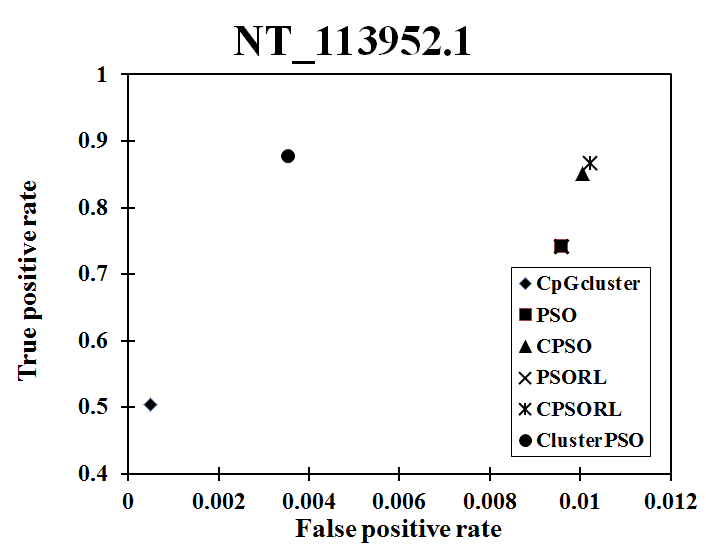

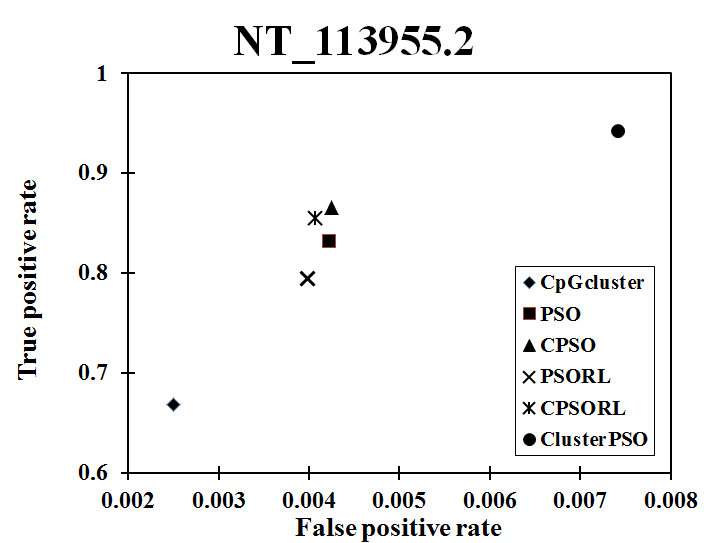
**


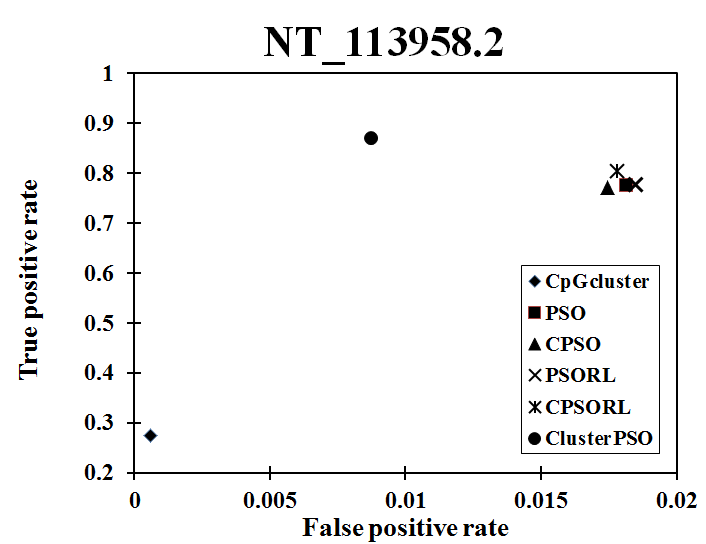

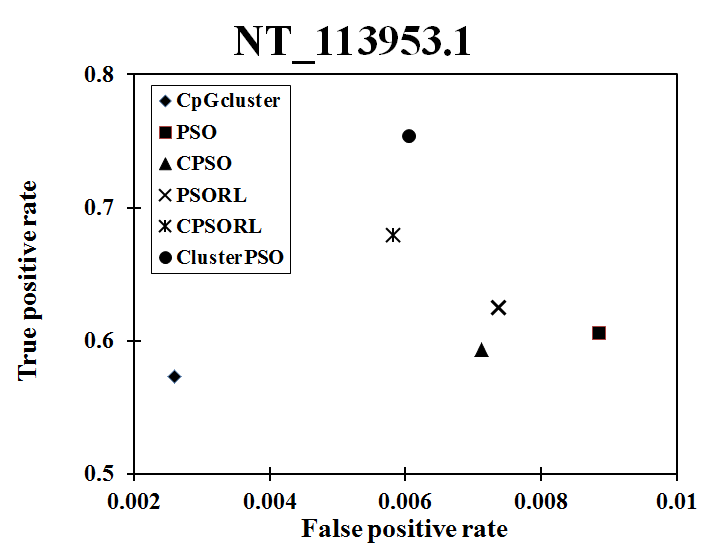


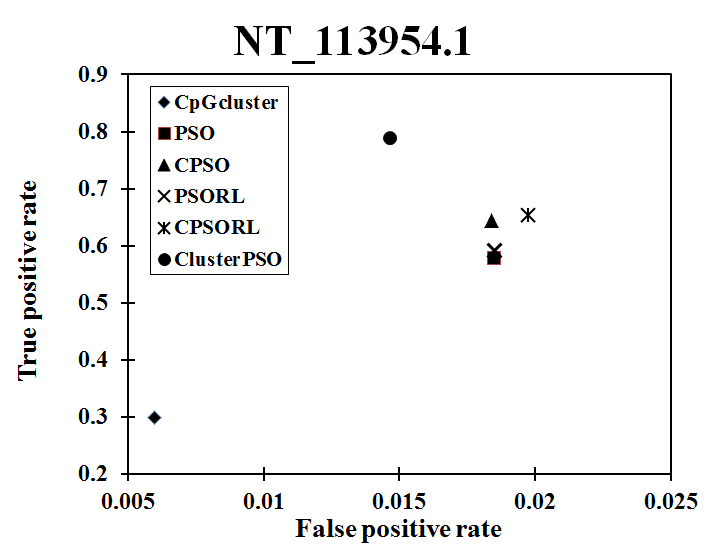

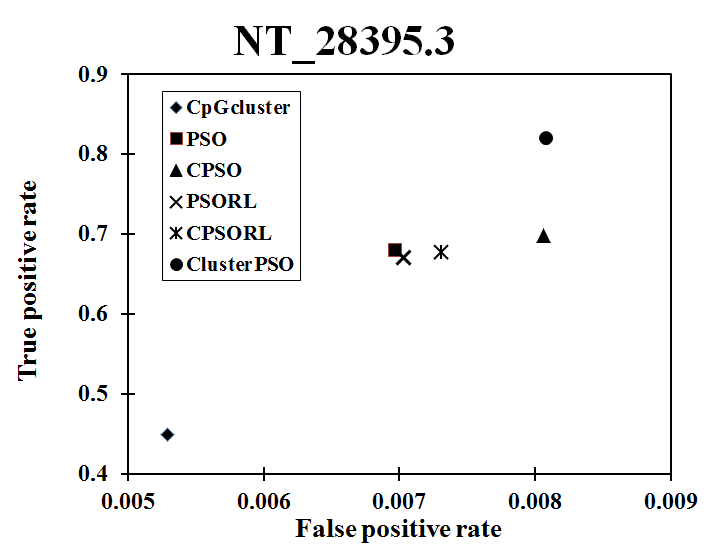


**Figure C.** XY charts comparing the true positive and false positive rates amongst the six methods for six contig sequences. The XY charts for the true and false positive rates are shown for Cluster, PSO, CPSO, PSORL, CPSORL and ClusterPSO to compare their relative accuracy rates in the six contig sequences.

**Figure D.** Box plot comparing the stability of five methods in six contig sequences. The five PSO-based methods use the same seed values for each test, generating the same random values for all PSO-based methods. The number of iterations is 1000 runs. The horizontal axis represents the scale value of the corresponding performance measurement. In the box plot, the boundary of the box closest to zero represents the 25^th^ percentile, a line within the box marks the median, and the boundary of the box farthest from zero represents the 75^th^ percentile. Error bars near the right and left of the boxes respectively indicate the 90^th^ and 10^th^ percentiles. The circles represent the outlier that is an observed point distant from other observations.

**Sensitivity of NT_113953.1**


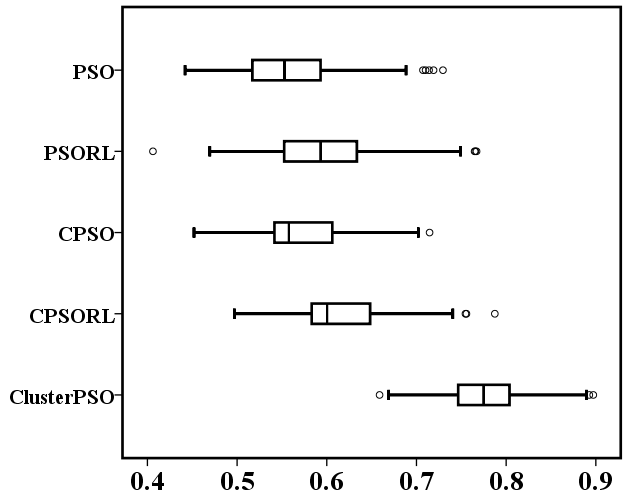


**Sensitivity of NT_113952.1**


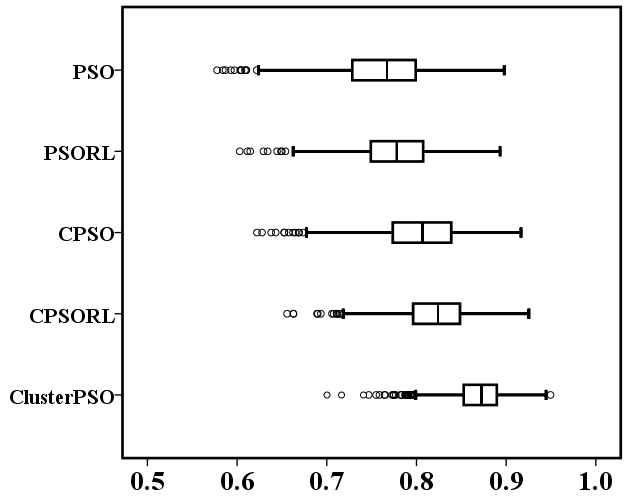


**Sensitivity of NT_113958.2**


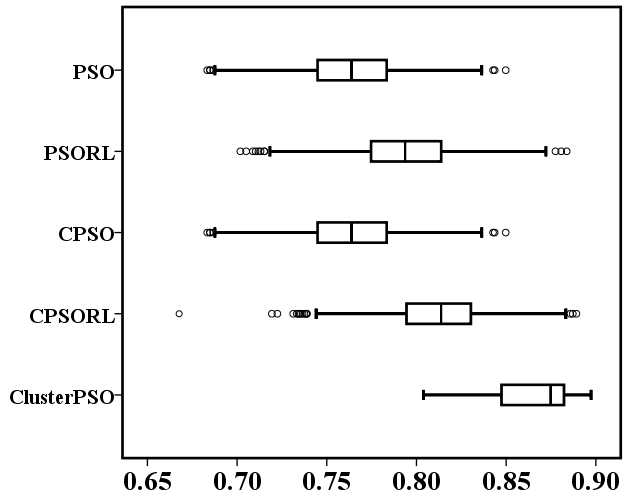


**Sensitivity of NT_028395.3**


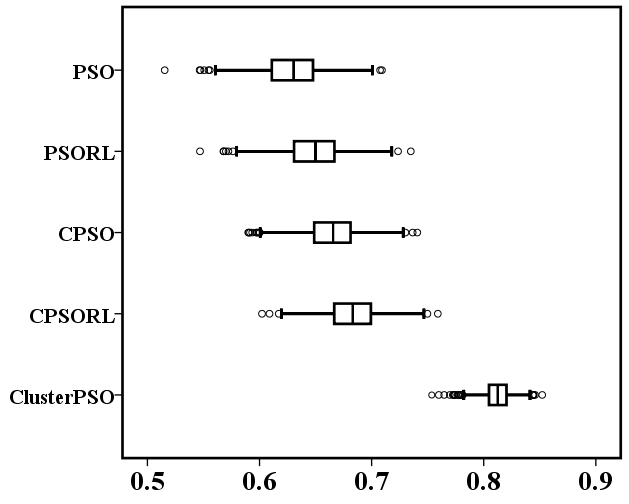


**Sensitivity of NT_113955.2**


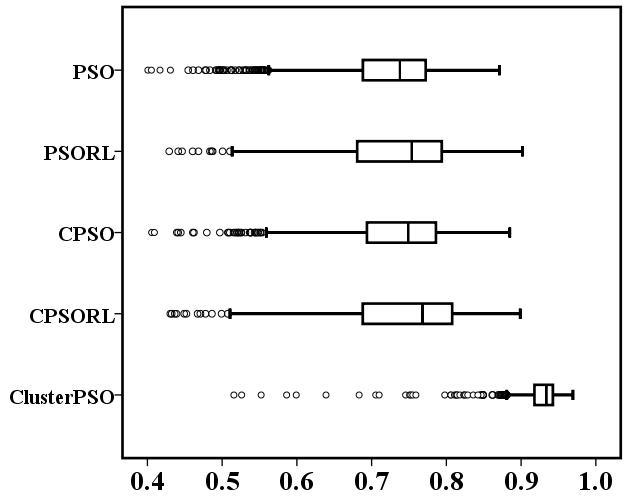


**Sensitivity of NT_113954.1**


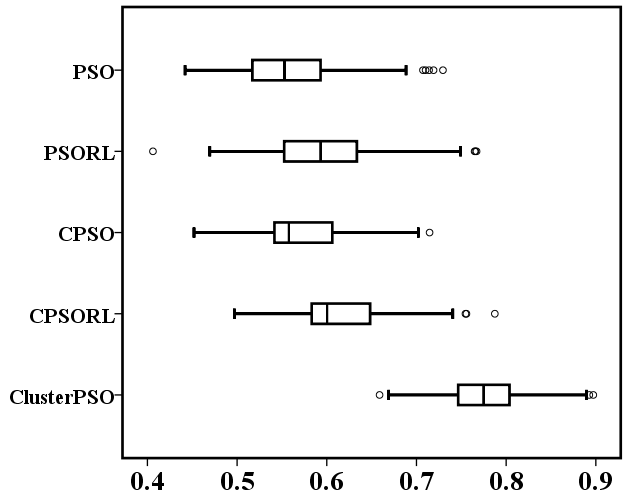


**Specificity of NT_113953.1**


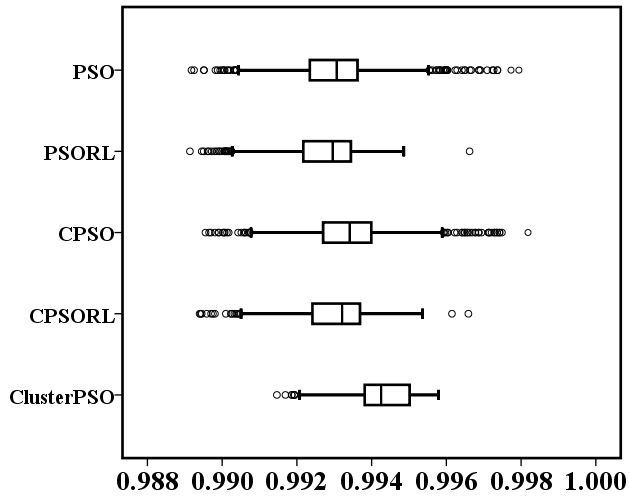


**Specificity of NT_113952.1**


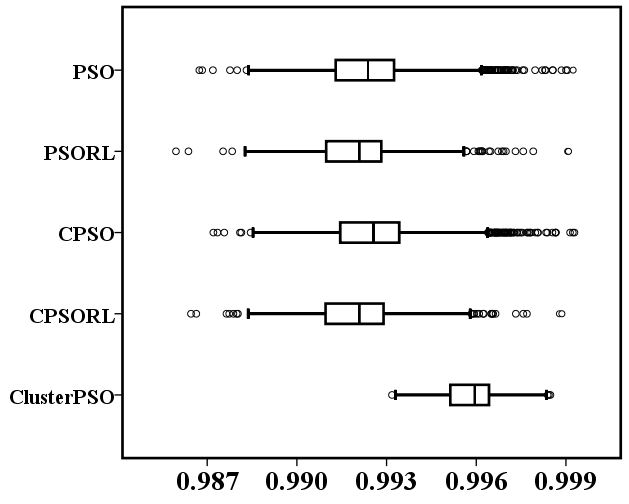


**Specificity of NT_113958.2**


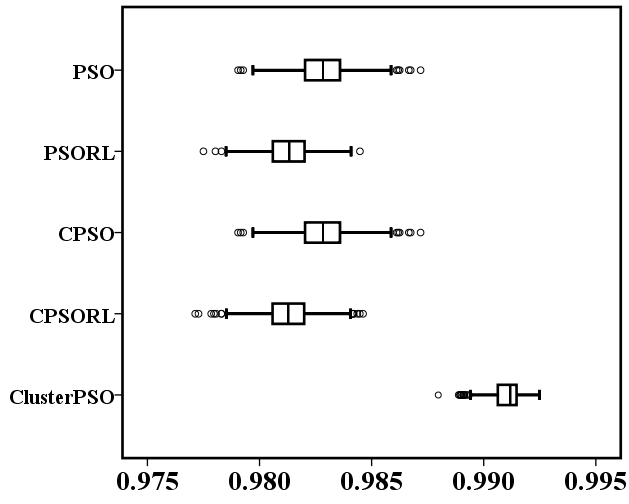


**Specificity of NT_028395.3**


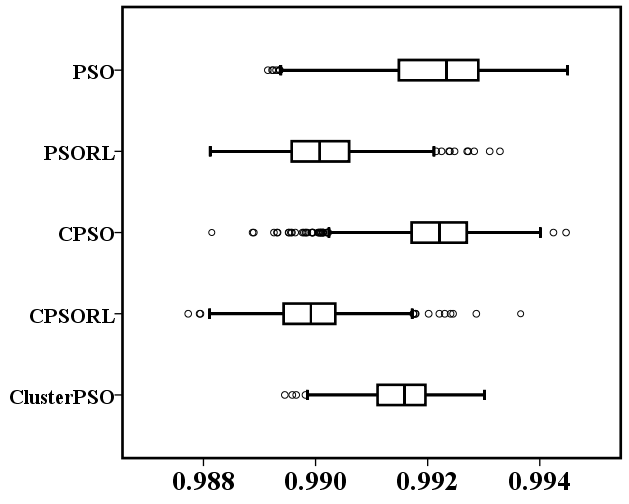


**Specificity of NT_113955.2**


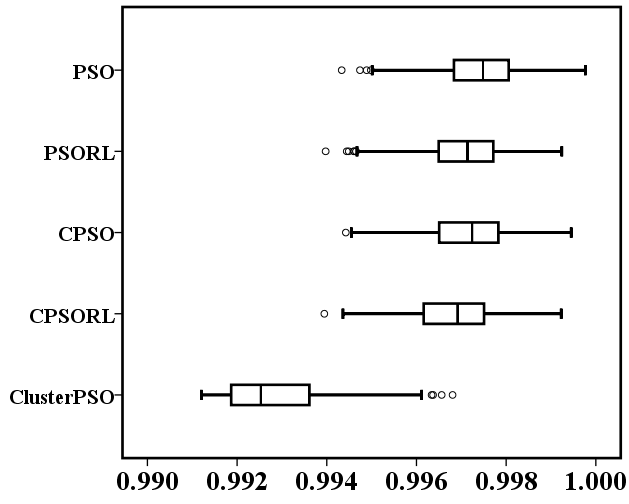


**Specificity of NT_113954.1**


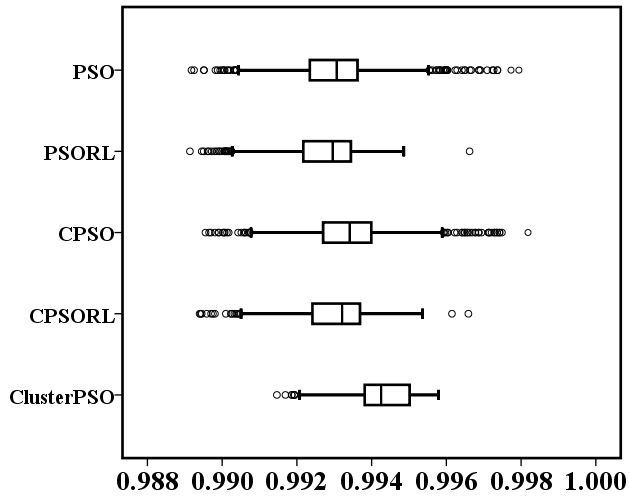


**Accuracy of NT_113953.1**


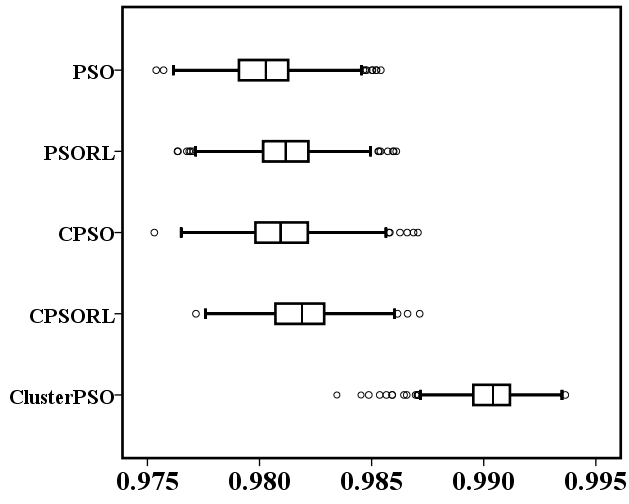


**Accuracy of NT_113952.1**


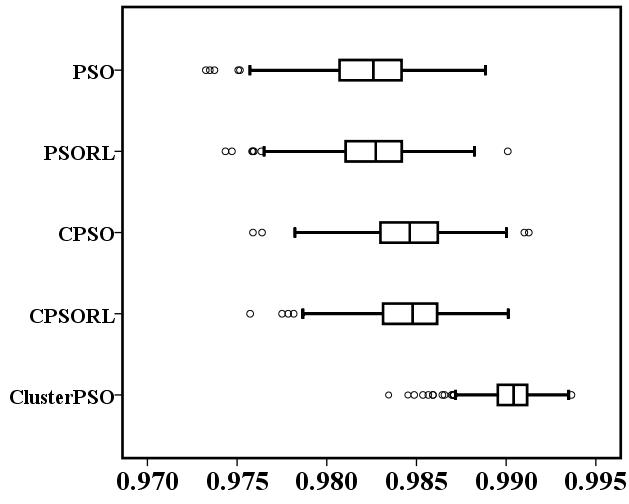


**Accuracy of NT_113958.2**


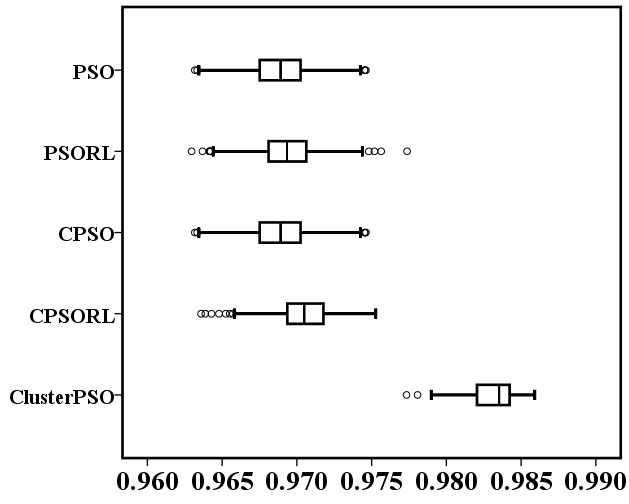


**Accuracy of NT_028395.3**


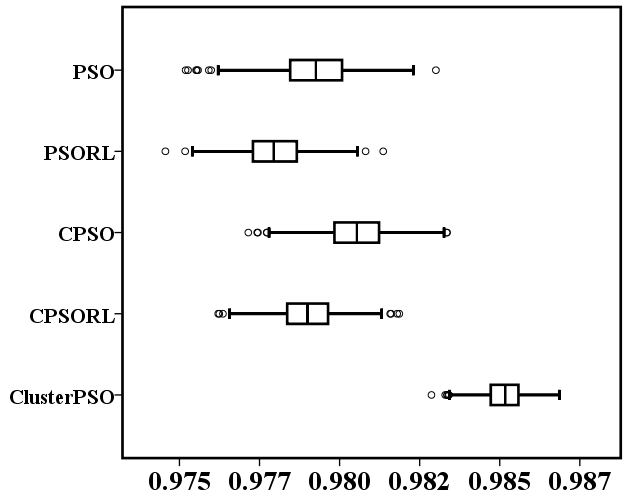


**Accuracy of NT_113955.2**


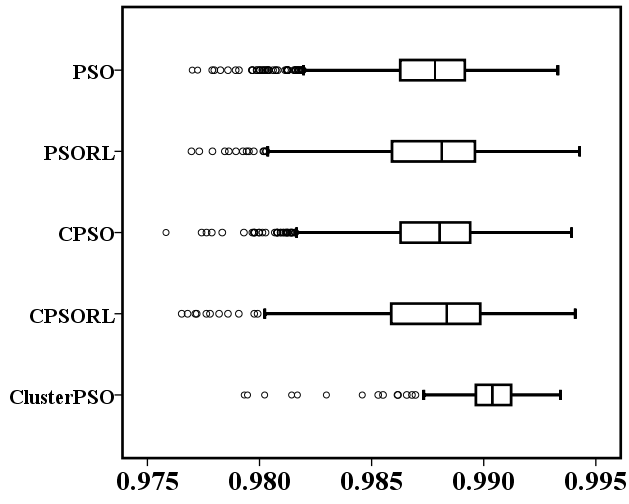


**Accuracy of NT_113954.1**


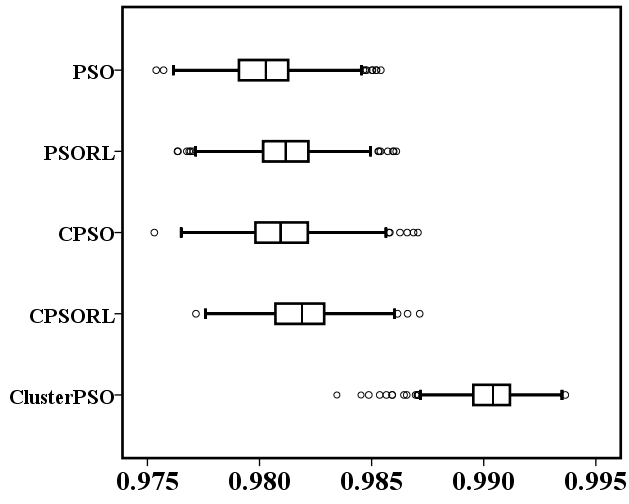


**Performance coefficient of NT_113953.1**


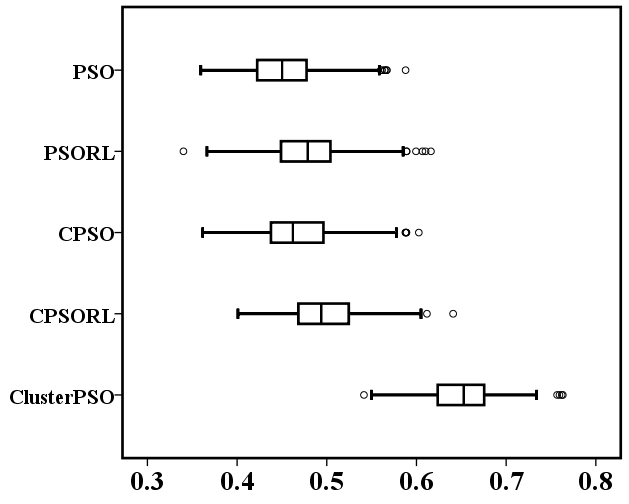


**Performance coefficient of NT_113952.1**


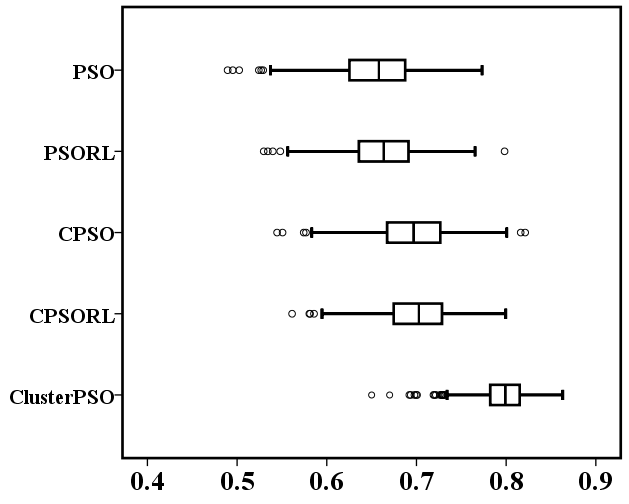


**Performance coefficient of NT_113958.2**


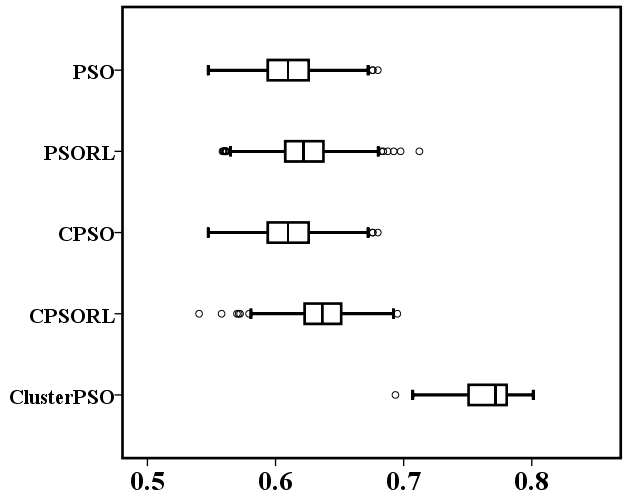


**Performance coefficient of NT_028395.3**


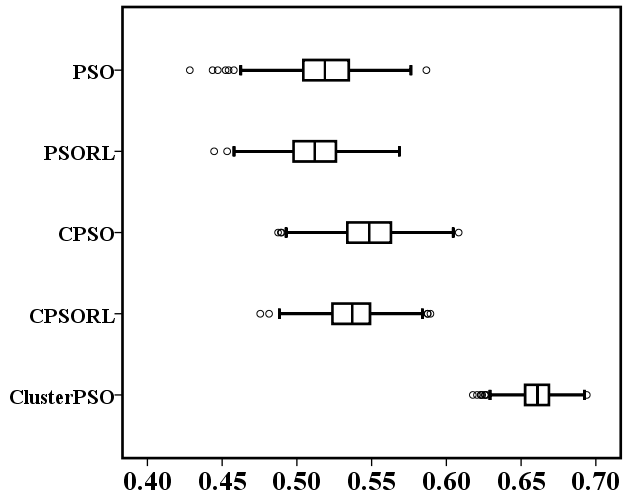


**Performance coefficient of NT_113955.2**


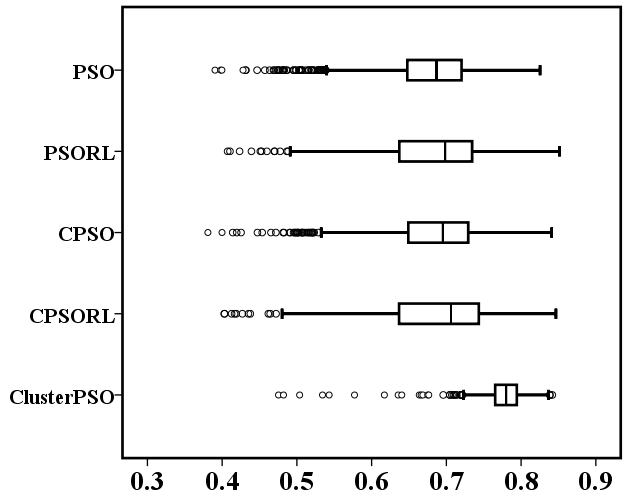


**Performance coefficient of NT_113954.1**


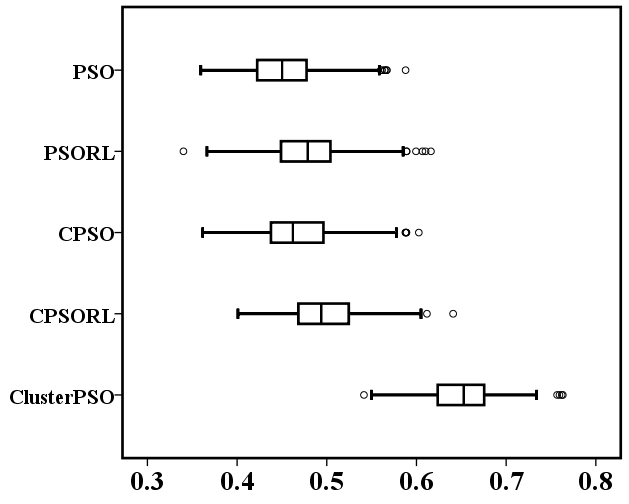


**Correlation coefficient of NT_113953.1**


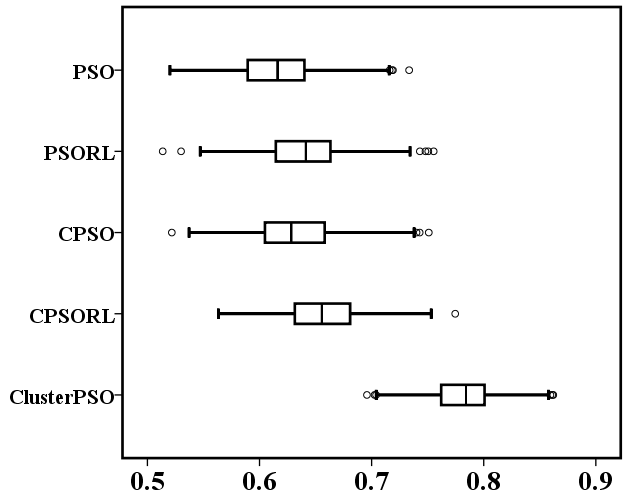


**Correlation coefficient of NT_113952.1**


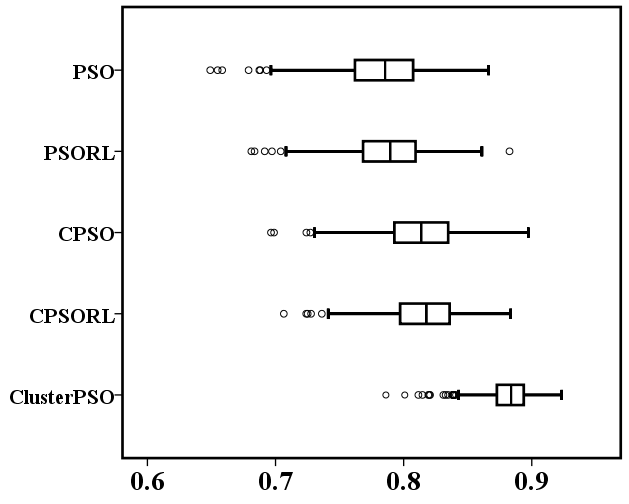


**Correlation coefficient of NT_113958.2**


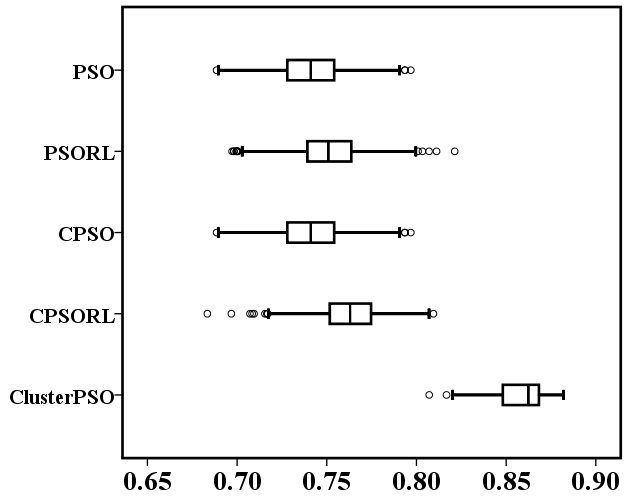


**Correlation coefficient of NT_028395.3**


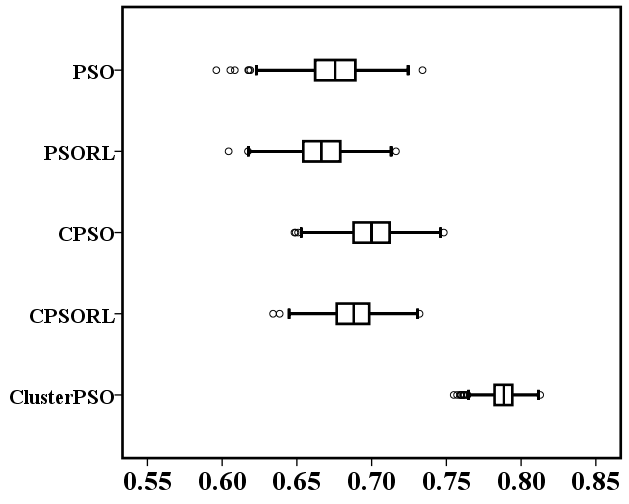


**Correlation coefficient of NT_113955.2**


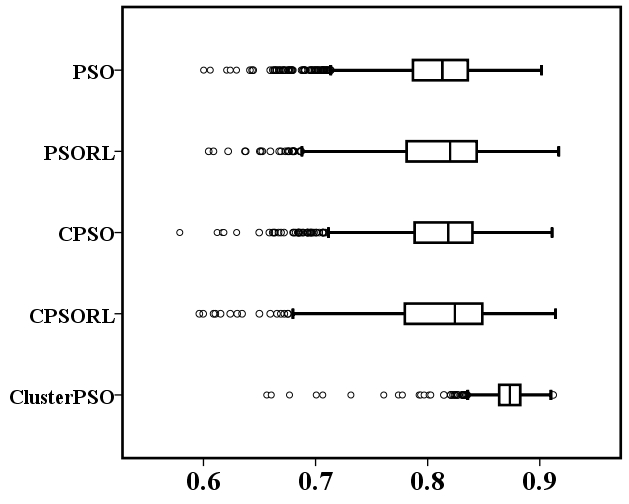


**Correlation coefficient of NT_113954.1**


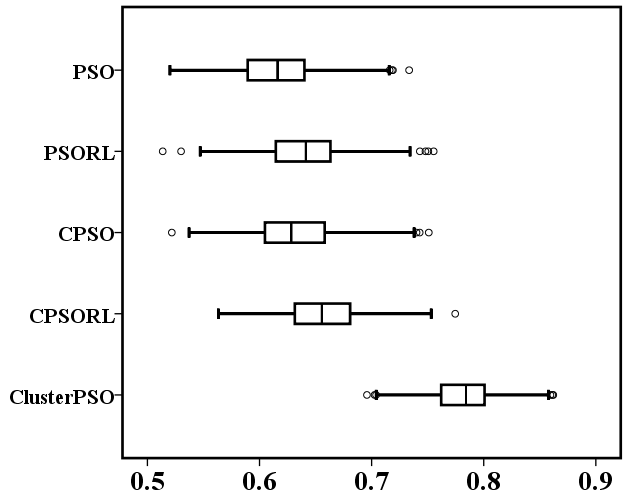


**Figure E.** Box plot of the O/E ratio for each interval length in the human genome.

| **Chromosome 1**  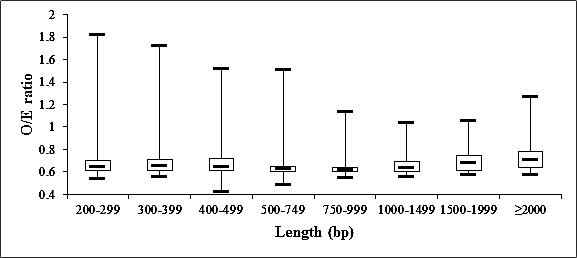 |
| --- |
| **Chromosome 2**  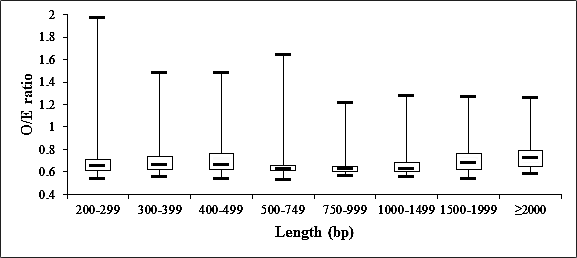 |
| **Chromosome 3**  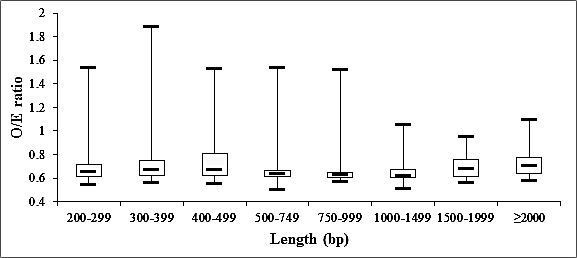 |

| **Chromosome 4**  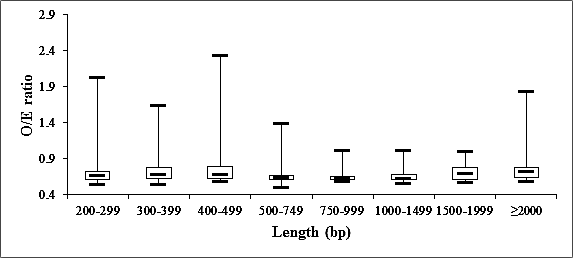 |
| --- |
| **Chromosome 5**  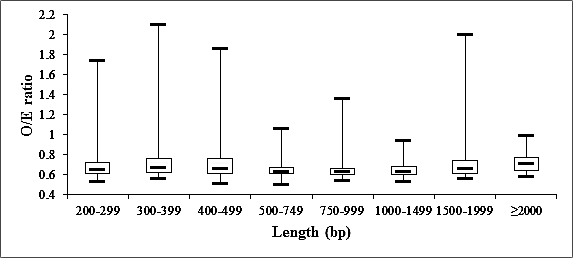 |
| **Chromosome 6**  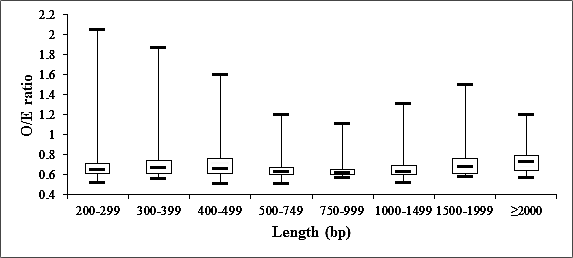 |

| **Chromosome 7**  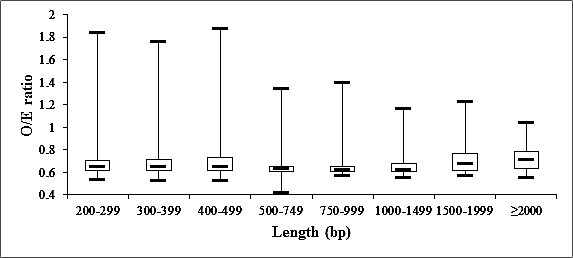 |
| --- |
| **Chromosome 8**  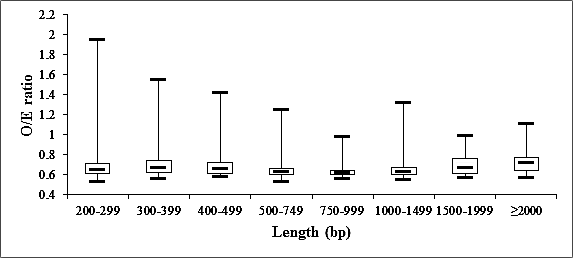 |
| **Chromosome 9**  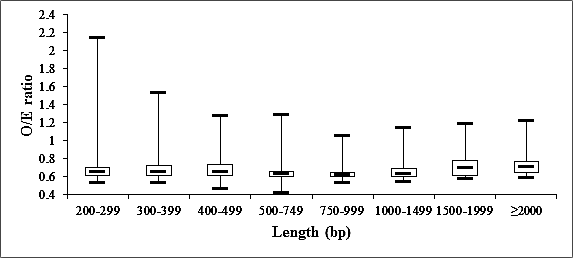 |

| **Chromosome 10**  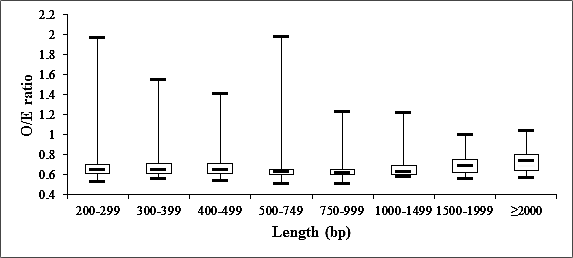 |
| --- |
| **Chromosome 11**  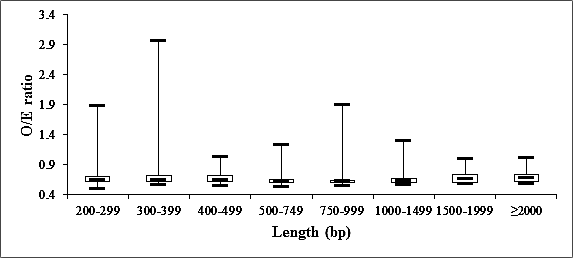 |
| **Chromosome 12**  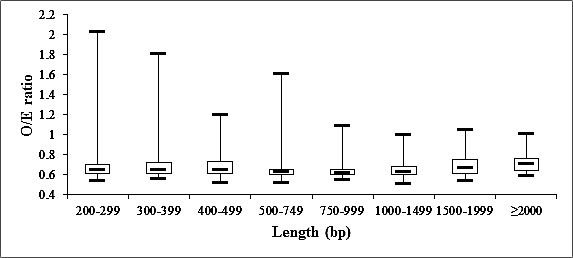 |

| **Chromosome 13**  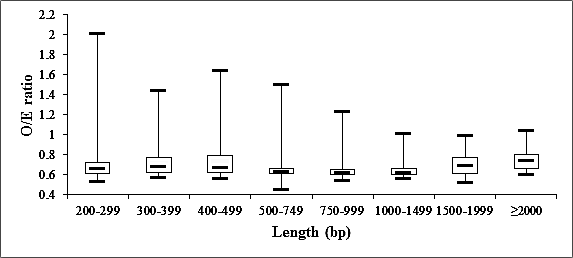 |
| --- |
| **Chromosome 14**  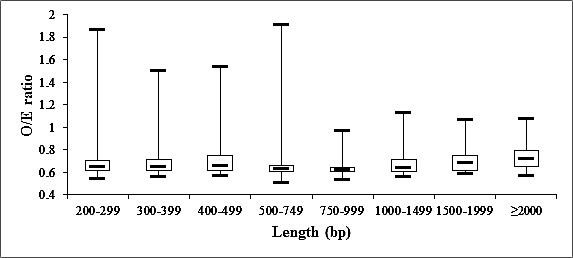 |
| **Chromosome 15**  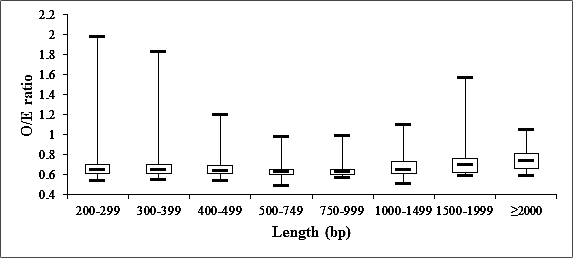 |

| **Chromosome 16**  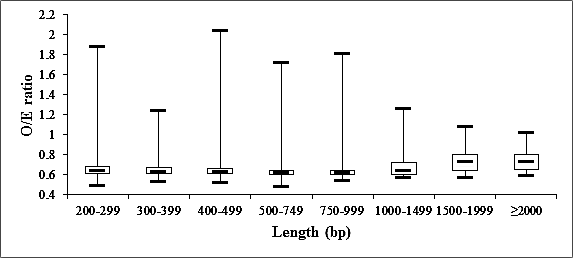 |
| --- |
| **Chromosome 17**  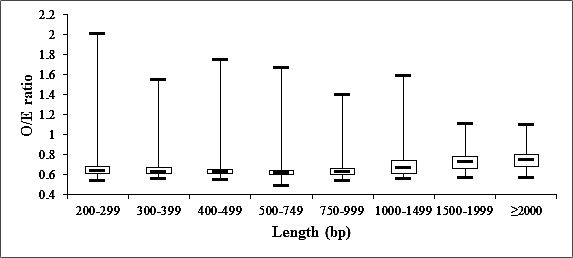 |
| **Chromosome 18**  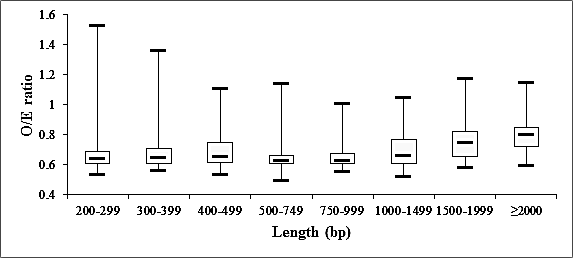 |
| **Chromosome 19**  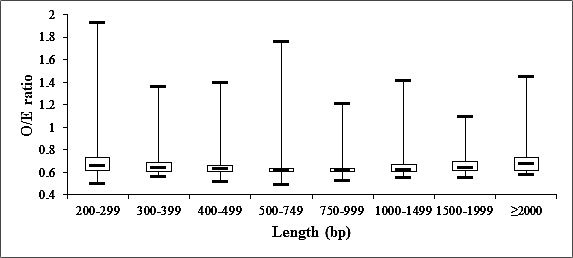 |
| **Chromosome 20**  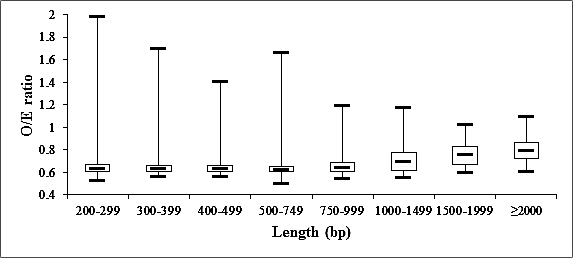 |
| **Chromosome 21**  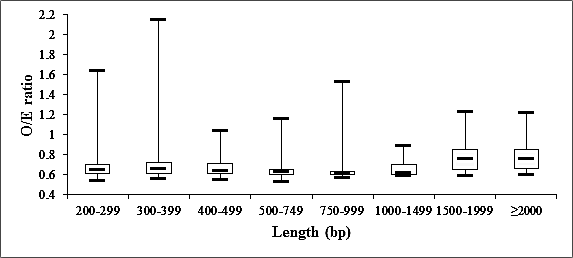 |
| **Chromosome 22**  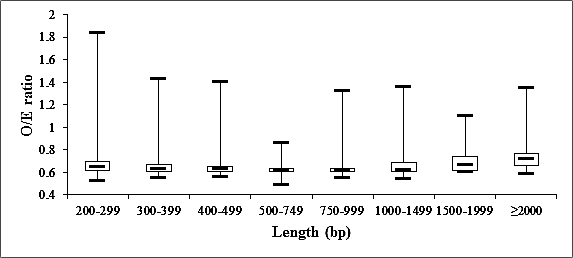 |
| **Chromosome X**  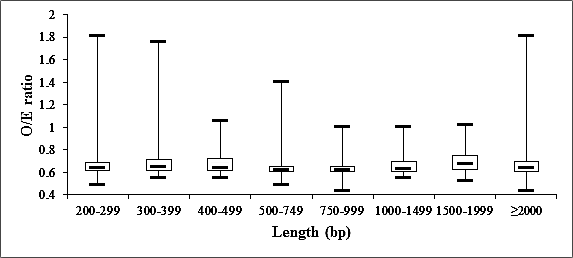 |
| **Chromosome Y**  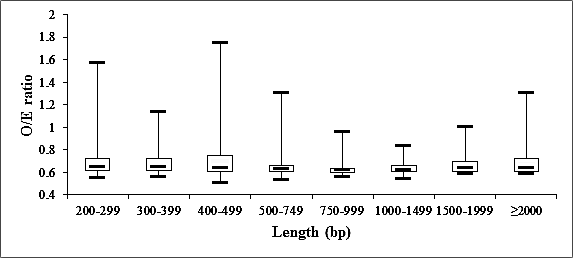 |

**Table A.** Number of CpG islands located in gene regions identified with CPSORL and ClusterPSO

| Chr. | Contig | GC% (Average) | |  | CpG island length | |  | CpG island number^a^ | |  |
| --- | --- | --- | --- | --- | --- | --- | --- | --- | --- | --- |
|  |  | CPSORL | ClusterPSO |  | CPSORL | ClusterPSO |  | CPSORL | ClusterPSO |  |
| 21 |  |  |  |  |  |  |  |  |  |  |
|  | NT_113952.1 | 54.34 | 54.31 |  | 8,537 | 8,658 |  | 12 | 15 |  |
|  | NT_113955.2 | 53.04 | 52.29 |  | 10,023 | 11,404 |  | 15 | 14 |  |
|  | NT_113958.2 | 57.01 | 58.89 |  | 14,470 | 13,516 |  | 19 | 24 |  |
|  | NT_113953.1 | 50.92 | 52.27 |  | 3,998 | 3,831 |  | 8 | 9 |  |
|  | NT_113954.1 | 54.53 | 56.47 |  | 6,174 | 5,931 |  | 10 | 17 |  |
| 22 |  |  |  |  |  |  |  |  |  |  |
|  | NT_028395.3 | 55.40 | 55.84 |  | 24,649 | 22,780 |  | 38 | 51 |  |

^a^The CpG island number column is the number of detected CpG islands in each contig.

**Table B.** Performance measurement of ClusterPSO and CPSORL for all chromosomes in the human genome

|  | CPSORL | | | | |  | ClusterPSO | | | | |
| --- | --- | --- | --- | --- | --- | --- | --- | --- | --- | --- | --- |
| Chr.^a^ | SN | SP | ACC | PC | CC |  | SN | SP | ACC | PC | CC |
| 1 | 67.45 | 99.02 | 97.77 | 54.67 | 69.62 |  | 79.15 | 99.02 | 98.22 | 64.02 | 77.14 |
| 2 | 65.94 | 99.22 | 98.12 | 53.68 | 69.02 |  | 78.80 | 99.18 | 98.51 | 63.63 | 77.01 |
| 3 | 67.07 | 99.31 | 98.36 | 54.63 | 69.92 |  | 79.04 | 99.24 | 98.64 | 63.18 | 76.76 |
| 4 | 66.68 | 99.36 | 98.43 | 54.62 | 69.98 |  | 77.16 | 99.31 | 98.68 | 62.37 | 76.14 |
| 5 | 66.40 | 99.23 | 98.19 | 53.65 | 69.00 |  | 78.76 | 99.18 | 98.54 | 62.95 | 76.52 |
| 6 | 67.36 | 99.15 | 98.06 | 54.37 | 69.52 |  | 79.59 | 99.11 | 98.44 | 63.59 | 76.95 |
| 7 | 66.43 | 98.93 | 97.52 | 53.70 | 68.69 |  | 77.51 | 98.96 | 98.03 | 63.02 | 76.29 |
| 8 | 66.22 | 99.21 | 98.11 | 53.91 | 69.2 |  | 77.17 | 99.18 | 98.45 | 62.41 | 76.05 |
| 9 | 65.26 | 99.06 | 97.73 | 53.01 | 68.26 |  | 78.62 | 99.10 | 98.32 | 64.16 | 77.30 |
| 10 | 64.97 | 99.06 | 97.72 | 52.82 | 68.10 |  | 77.71 | 99.06 | 98.22 | 63.11 | 76.46 |
| 11 | 66.74 | 99.04 | 97.79 | 53.85 | 68.95 |  | 78.76 | 99.01 | 98.23 | 63.20 | 76.54 |
| 12 | 66.91 | 98.99 | 97.64 | 54.38 | 69.33 |  | 78.00 | 98.98 | 98.09 | 63.27 | 76.51 |
| 13 | 66.81 | 99.41 | 98.54 | 54.99 | 70.35 |  | 77.47 | 99.37 | 98.79 | 62.96 | 76.64 |
| 14 | 66.47 | 99.21 | 98.13 | 53.96 | 69.24 |  | 78.78 | 99.19 | 98.52 | 63.71 | 77.08 |
| 15 | 66.37 | 99.12 | 97.95 | 53.61 | 68.84 |  | 79.53 | 99.13 | 98.43 | 64.36 | 77.51 |
| 16 | 65.32 | 98.44 | 96.41 | 52.61 | 67.17 |  | 78.79 | 98.60 | 97.40 | 64.76 | 77.22 |
| 17 | 65.32 | 98.44 | 96.43 | 52.61 | 67.17 |  | 80.32 | 98.19 | 96.74 | 66.61 | 78.18 |
| 18 | 65.62 | 99.23 | 98.14 | 53.51 | 68.90 |  | 81.77 | 99.28 | 98.71 | 67.42 | 79.88 |
| 19 | 69.09 | 96.46 | 93.03 | 55.40 | 67.39 |  | 71.35 | 97.14 | 93.91 | 59.48 | 71.24 |
| 20 | 65.22 | 98.74 | 97.06 | 52.69 | 67.60 |  | 79.63 | 98.80 | 97.83 | 64.87 | 77.55 |
| 21 | 66.81 | 99.18 | 98.03 | 54.73 | 69.86 |  | 78.67 | 99.19 | 98.46 | 64.55 | 77.66 |
| 22 | 65.43 | 98.55 | 96.54 | 53.41 | 67.96 |  | 74.08 | 98.62 | 97.13 | 61.00 | 74.28 |
| X | 65.97 | 99.25 | 98.21 | 53.53 | 68.93 |  | 82.06 | 99.26 | 98.72 | 66.66 | 79.36 |
| Y | 64.91 | 99.55 | 98.99 | 50.80 | 66.91 |  | 79.10 | 99.64 | 99.31 | 64.78 | 78.28 |
|  |  |  |  |  |  |  |  |  |  |  |  |
| Ave.^b^ | 66.28 | 98.97 | 97.62 | 53.71 | 68.75 |  | 78.41 | 98.99 | 98.10 | 63.75 | 76.86 |

^a^ Human chromosomes (NCBI.36). ^b^Ave.: Average of measurement values. SN = Sensitivity, SP = Specificity, ACC = Accuracy , PC = Performance coefficient, CC = Correlation coefficient

**Table C.** Number of detection CpG islands overlapping on true CpG islands for CpGcluster, CPSORL and ClusterPSO for all chromosomes in the human genome

|  |  | CpGcluster | | |  | CPSORL | |  | ClusterPSO | |
| --- | --- | --- | --- | --- | --- | --- | --- | --- | --- | --- |
| Chr | Number of  true CpG island | Number of  detection island | Additional^a^ | |  | Number of  detection island | Additional |  | Number of  detection island | Additional |
| 1 | 25,035 | 15,345 | | 11,986 |  | 15,394 | 10,597 |  | 25,035 | 6,859 |
| 2 | 20,833 | 13,711 | | 9,626 |  | 12,582 | 9,180 |  | 20,833 | 5,698 |
| 3 | 15,321 | 10,704 | | 6,515 |  | 9,386 | 6,517 |  | 15,321 | 4,008 |
| 4 | 13,974 | 10,627 | | 5,256 |  | 8,846 | 5,827 |  | 13,974 | 3,722 |
| 5 | 14,582 | 10,385 | | 5,904 |  | 9,002 | 6,207 |  | 14,582 | 3,848 |
| 6 | 15,054 | 10,272 | | 6,384 |  | 9,891 | 4,118 |  | 15,054 | 3,982 |
| 7 | 17,866 | 11,388 | | 8,186 |  | 11,079 | 6,625 |  | 17,866 | 5,076 |
| 8 | 12,582 | 8,506 | | 5,671 |  | 7,678 | 5,527 |  | 12,582 | 3,598 |
| 9 | 13,858 | 8,697 | | 6,666 |  | 8,763 | 5,347 |  | 13,858 | 3,719 |
| 10 | 13,867 | 7,910 | | 7,275 |  | 8,215 | 6,394 |  | 13,867 | 4,046 |
| 11 | 13,229 | 8,568 | | 6,440 |  | 8,118 | 5,661 |  | 13,229 | 3,655 |
| 12 | 14,681 | 6,187 | | 4,500 |  | 9,039 | 6,260 |  | 14,681 | 3,989 |
| 13 | 7,976 | 4,626 | | 2,809 |  | 4,959 | 3,269 |  | 7,976 | 2,128 |
| 14 | 9,099 | 5,713 | | 4,347 |  | 5,543 | 3,858 |  | 9,099 | 2,471 |
| 15 | 9,330 | 5,025 | | 4,969 |  | 5,660 | 3,972 |  | 9,330 | 2,633 |
| 16 | 14,017 | 6,005 | | 9,255 |  | 8,394 | 6,366 |  | 14,017 | 3,810 |
| 17 | 16,569 | 5,940 | | 11,753 |  | 10,062 | 7,275 |  | 16,569 | 4,207 |
| 18 | 6,562 | 4,415 | | 2,794 |  | 3,997 | 3,117 |  | 6,562 | 1,601 |
| 19 | 17,512 | 5,131 | | 13,402 |  | 11,294 | 7,003 |  | 17,512 | 6,389 |
| 20 | 8,192 | 3,947 | | 4,894 |  | 4,774 | 3,845 |  | 8,192 | 2,278 |
| 21 | 4,547 | 2,816 | | 2,304 |  | 2,842 | 1,862 |  | 4,547 | 1,135 |
| 22 | 8,215 | 2,179 | | 6,540 |  | 4,864 | 3,647 |  | 8,215 | 2,733 |
| X | 12,628 | 9,202 | | 4,880 |  | 7,876 | 5,260 |  | 12,628 | 2,962 |
| Y | 2,367 | 1,913 | | 814 |  | 1,574 | 965 |  | 2,367 | 561 |
| Totle | 307,896 | 179,212 | | 153,170 |  | 189,832 | 128,699 |  | 25,035 | 6,859 |
| Avg. | 12,829 | 7,467 | | 6,382 |  | 7,910 | 5,362 |  | 20,833 | 5,698 |

^a^Additional is the number of non-true CpG islands.
